# The role of inflammatory biomarkers in the development and progression of pre-eclampsia: a systematic review and meta-analysis

**DOI:** 10.3389/fimmu.2023.1156039

**Published:** 2023-05-30

**Authors:** Xiaohan Guan, Yanwen Fu, Yixin Liu, Mingxuan Cui, Caishun Zhang, Qing Zhang, Chunmei Li, Jian Zhao, Chaofan Wang, Jiarun Song, Jing Dong

**Affiliations:** ^1^ School of Basic Medicine, Clinical Medicine Department of Medical College, Qingdao University, Qingdao, Shandong, China; ^2^ Special Medicine Department, Medical College, Qingdao University, Qingdao, Shandong, China; ^3^ School of Public Health, Medical College, Qingdao University, Qingdao, Shandong, China; ^4^ Physiology Department, Medical College, Qingdao University, Qingdao, Shandong, China

**Keywords:** pre-eclampsia, biomarkers, inflammation, meta-analysis, IL-6, IL-10, TNF

## Abstract

**Background:**

Pre-eclampsia (PE) is a pregnancy complication associated with maternal and fetal morbidity and mortality. Among the potential pathogenesis discussed, inflammation is considered an essential initiator of PE. Previous studies have compared the levels of various inflammatory biomarkers that indicate the existence of PE; however, the relative levels of pro-inflammatory and anti-inflammatory biomarkers and their dynamic changes during PE progression remain unclear. This knowledge is essential to explain the occurrence and progression of the disease.

**Objective:**

We aimed to identify the relationship between inflammatory status and PE using inflammatory biomarkers as indicators. We also discussed the underlying mechanism by which inflammatory imbalance contributes to PE by comparing the relative levels of pro-inflammatory and anti-inflammatory biomarkers. Furthermore, we identified additional risk factors for PE.

**Methods:**

We reviewed PubMed, Embase, and the Cochrane Library for articles published until 15^th^ September 2022. Original articles that investigated inflammatory biomarkers in PE and normal pregnancy were included. We selected healthy pregnant women as controls. The inflammatory biomarkers in the case and control groups were expressed as standardized mean differences and 95% confidence intervals using a random-effects model. Study quality was assessed using the Newcastle-Ottawa Scale. Publication bias was assessed using Egger’s test.

**Results:**

Thirteen articles that investigated 2,549 participants were included in this meta-analysis. Patients with PE had significantly higher levels of C-reactive protein (CRP), interleukin (IL)-4, IL-6, IL-8, IL-10, and tumor necrosis factor (TNF) than the controls. CRP and pro-inflammatory cytokine levels were higher than those of anti-inflammatory cytokines. Patients with gestational age > 34 weeks had significantly higher IL-6 and TNF levels. Patients with higher systolic blood pressure had significantly higher IL-8, IL-10, and CRP levels.

**Conclusion:**

Inflammatory imbalance is an independent risk factor for PE development. Impairment of the anti-inflammatory system is a crucial initiating factor for PE development. Failed autoregulation, manifested as prolonged exposure to pro-inflammatory cytokines, leads to PE progression. Higher levels of inflammatory biomarkers suggest more severe symptoms, and pregnant women after 34 weeks of gestation are more susceptible to PE.

## Introduction

Pre-eclampsia (PE) is a pregnancy complication associated with substantial maternal and fetal morbidity and mortality (1). It is new-onset hypertension that occurs after 20 weeks of gestation, complicated with proteinuria or other severe complications, including thrombocytopenia, impaired liver function, renal insufficiency, pulmonary edema, and new-onset headache unresponsive to medication and not accounted for by alternative diagnosis or visual symptoms (2).

PE is thought to progress in two stages: (1) abnormal placentation early in the first trimester and (2) maternal syndrome in the late second and third trimesters. Evidence suggests that the diseased placenta releases soluble toxic factors into the maternal circulation, resulting in inflammation (3). PE and inflammation can occur simultaneously or sequentially.

Research on the pathophysiology of PE has led to the discovery of changes in the levels of circulating factors released during pre-eclamptic pregnancies (4). Biomarkers can help clarify the likelihood of PE when the clinical picture is uncertain (5). Moreover, inflammatory biomarkers have become potential therapeutic targets for intervention at all stages of the disease process (6).

C-reactive protein (CRP) is a biomarker of systemic inflammation (7). CRP regulates inflammatory progression and increases the incidence of chronic inflammatory diseases, including cardiovascular diseases (8). The relative levels of pro-inflammatory and anti-inflammatory cytokines contribute to the balance of the inflammatory system, disturbances of which lead to PE. Increased pro-inflammatory cytokines such as interleukin (IL)-6, IL-8, and tumor necrosis factor (TNF) are related to immune activity against the fetus, elevated blood pressure, and target organ damage (8–11). Anti-inflammatory cytokines, such as IL-4 and IL-10, can attenuate the generation of pro-inflammatory cytokines, alleviate inflammation, and reduce blood pressure (10, 12–14).

A connection between PE and inflammation has been deduced; however, inflammatory biomarker levels vary across studies. Previous studies have provided inconclusive data; some reported an increase, and others a decrease in IL-10 levels (15). The situation is similar for pro-inflammatory biomarkers; some studies have reported significant increases in pro-inflammatory biomarkers (16, 17), whereas others have reported no significant changes (18, 19). These discrepancies may be due to differences in patient characteristics, especially disease severity and comorbidities, including diabetes, chronic kidney disease, and autoimmune disorders (18, 20). In addition, studies have shown that biomarker levels depend on gestational age (GA), and the relative risk of PE varies with GA (21, 22). Furthermore, different sample characteristics (i.e., detection methods and sample types) may cause variations in biomarker levels (15).

Many theories regarding the underlying mechanism of PE have been discussed, and inflammatory imbalance is now considered an important factor. Although several studies have focused on the changes in inflammatory biomarkers during PE, their results vary. These variations may be due to the characteristics of the patients, samples, or the detection methods used. Therefore, a systemic analysis of these studies is needed.

We selected CRP, pro-inflammatory cytokines (IL-6, TNF, and IL-8), and anti-inflammatory cytokines (IL-4 and IL-10) as our target biomarkers to determine the role of inflammatory status in PE development and progression and to identify factors that influence biomarker levels.

## Materials and methods

This meta-analysis was based on the Preferred Reporting Items for Systematic Reviews and Meta-analyses guidelines (23).

### Data sources and search strategy

Two investigators independently searched PubMed, Embase, and Cochrane Library databases. We used a combination of key words and Medical Subject Headings terms as our search strategy: (pre-eclampsia OR pregnancy toxemias OR pregnancy toxemia OR edema-proteinuria-hypertension gestosis OR edema proteinuria hypertension gestosis OR EPH complex OR EPH toxemias OR EPH toxemia OR EPH gestosis) AND (inflammation OR inflammations OR innate inflammatory response OR innate inflammatory responses) AND (cohort studies OR case-control studies OR comparative study OR risk factors OR cohort OR compared OR groups OR case control OR multivariate). The complete search strategy is presented in (**Supplementary Table 1**).

### Selection criteria

We included publications that met the following criteria: 1) studies that compared healthy pregnant women (control) with patients with PE; 2) studies that used PE definition that met the recent American College of Obstetrics and Gynecology (ACOG) criteria; 3) studies that included at least one of the following parameters: IL-6, TNF, IL-8, IL-4, IL-10, and CRP; 4) studies that used maternal blood samples; 5) studies that used samples obtained before or at delivery; 6) studies published in English; 7) studies published after 2010.

The exclusion criteria were as follows: 1) studies that used other kinds of samples, including placental tissue and fetal blood; 2) studies with unclear PE definition; 3) studies with inaccessible full text or required data; 4) studies that included participants with chronic hypertension, chronic renal disease or preexisting proteinuria, type I and II diabetes, malignancy, gastrointestinal disease (Crohn’s disease, colitis ulcerosa), autoimmune disorders, acute systemic inflammation or fever; 5) reviews, meta-analysis, case reports, letters and comments, meeting abstracts, and posters.

We selected patients according to the most recent ACOG diagnostic criteria (1): hypertension occurring after 20 weeks of gestation: systolic blood pressure (SBP) ≥ 140 mmHg or diastolic blood pressure (DBP) ≥ 90 mmHg on two occasions at least 4 hours apart in a woman with a previously normal blood pressure; (2) A. proteinuria: urinary protein ≥ 300 mg in a 24-hour collection, protein/creatinine ratio of ≥ 0.3 or +2 by urine dipstick if quantitative methods are unavailable; B. absence of proteinuria but with the new-onset of any of the severe features (except the blood pressure standard). Severe features include SBP ≥ 160 mm Hg or DBP ≥ 110 mm Hg on two occasions at least 4 hours apart, thrombocytopenia, impaired liver function, renal insufficiency, pulmonary edema, and new-onset headache unresponsive to medication and not accounted for by alternative diagnoses or visual symptoms. Patients with severe features were diagnosed with severe pre-eclampsia (SPE), and others were considered to have mild pre-eclampsia (MPE) (2).

### Data extraction and quality assessment

Two authors independently extracted the data according to a predefined spreadsheet. The following data were obtained: study information (the article name and publication year), participants’ characteristics (country, PE definition, participation number, age, GA, body mass index (BMI), SBP, and DBP), and sample characteristics (sample type and analytical method).

The quality of each study was assessed using the Newcastle-Ottawa Scale. The quality evaluation criteria included (1) selection of case and control: A. case definition; B. representativeness of the cases; C. control selection; D. control definition comparability of case and control.; (2) Comparability: comparability of cases and controls based on the design or analysis.; (3) Exposure: A. ascertainment of exposure; B. same method of ascertainment for cases and controls; C. non-response rate. The assessment results were placed in **Supplementary Table 2**. Stars were assigned to each parameter ranging from 0 (lowest) to 9 (highest). Studies with a score ≥ 7 were considered high quality, and other studies were classified as having moderate quality.

### Data synthesis and analysis

All data were converted into mean values and standard deviations (24, 25). Patients with PE and controls were compared using a random-effects model. Continuous variables were analyzed using standard mean difference (SMD) and 95% confidence interval (CI). We used Cochran’s (chi-square) test to measure heterogeneity and the I² statistic to determine the extent of consistency: I² > 75% indicates a high level of inconsistency, > 50% is moderate, and < 25% is low (26). We used random-effect models to estimate pooled effect sizes. A two-sided p-value < 0.05 was considered statistically significant. Publication bias was assessed using Egger’s regression asymmetry test and inspection funnel plots (27). We conducted an influence analysis to determine the impact of a single study on the overall results.

Subgroup analyses were performed according to the test method, GA, sample size, age, BMI, SBP, and DBP. Subgroup analysis by GA was based on the following subtype diagnostic criteria: (1) early onset PE (EOP): ≤ 34 weeks of GA; (2) late-onset PE (LOP): > 34 weeks of GA (28–30). Subgroup analysis by SBP was based on PE severity (2).

All statistical analyses were performed using Stata Software (Version 17.0.0) and GraphPad Prism (GraphPad Prism 9.0.0 Macintosh Version by Software MacKiev).

## Results

### Literature search

**Figure 1:**
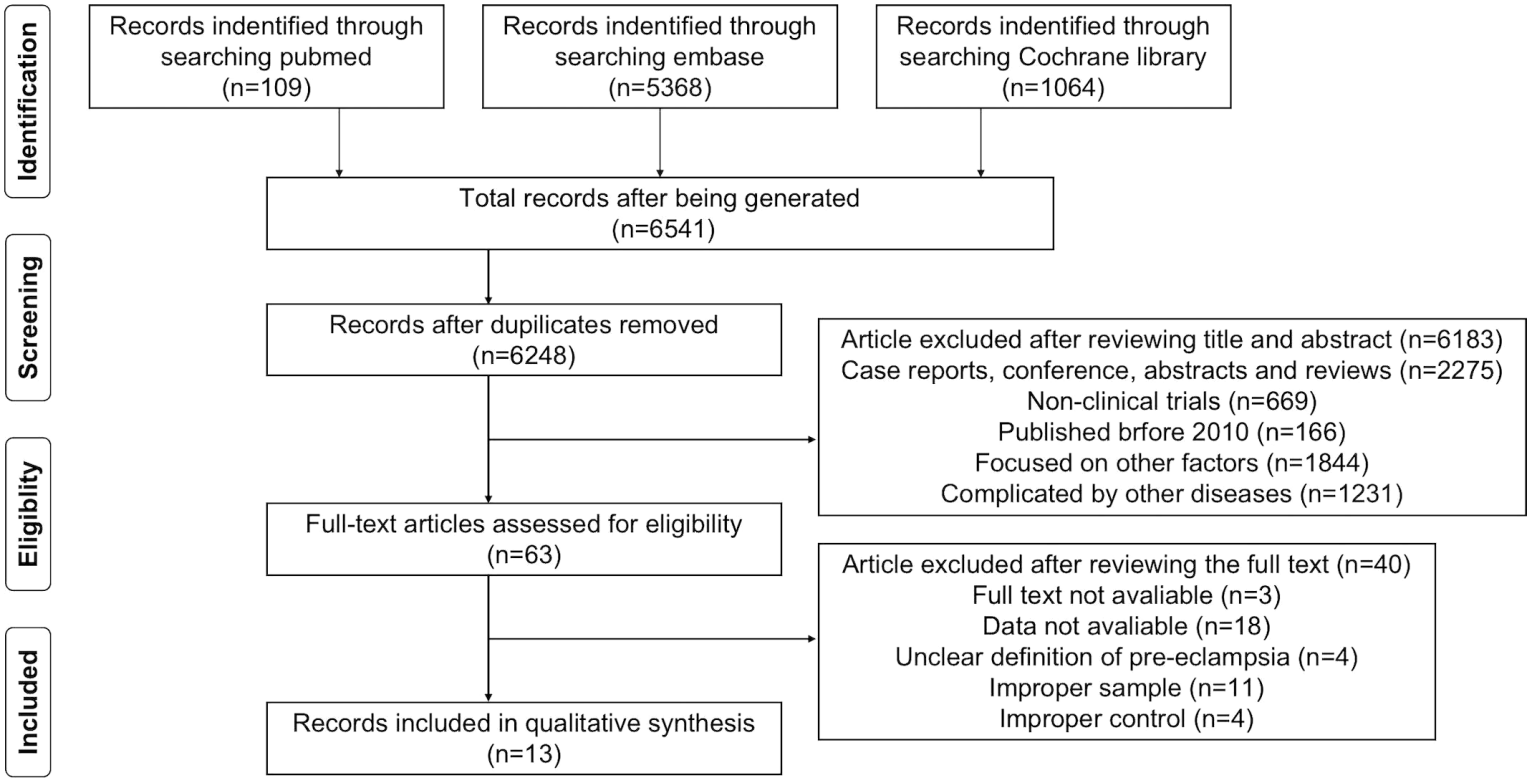
Flow chart of the literature search.

The details of the search are shown in **Figure 1**. We identified 6,541 potential reports from PubMed, Embase, and the Cochrane Library. We included all retrievable articles in an endnote database for examination. Finally, 13 studies met the selection criteria and were analyzed.

### Study characteristics

The study characteristics are presented in **Table 1**. We included 13 articles with 2,549 participants. All studies compared the biomarker levels between patients with PE and controls. Nine studies directly compared patients with PE with controls (16, 21, 31–37). Four studies classified patients with PE as severe or mild according to clinical manifestations. We also performed a cross-group comparison between the three groups (MPE vs. control, SPE vs. control, and MPE vs. SPE) (17, 38, 39). One study compared patients with SPE and control (40).

**Table 1:** Characteristics of included studies.

Author	Year	Country	N	Group set	Biomarkers		N	Age, y	Gestational Age, y	BMI	SBP	DBP
András Szarka et al.	2010	Hungary	120	PE vs. ctrl	IL-1β, IL-1ra, IL-2, IL-4, IL-6, IL-8, IL-10, IL-12p40, IL-12p70, IL-18, IFN-γ, TNF, IP-10, MCP-1, ICAM-1, VCAM-1, TGF-β1, CRP	PE	60	29 (26-32)^b^	37 (36-39)^b^	29.9 (26.9-33.3)^b^	162 (155-180)^b^	100 (97-110)^b^
						ctrl	60	29 (26-32)^b^	36 (36-37)^b^	25.8 (24.3-27.9)^b^	110 (107-120)^b^	70 (60-80)^b^
Gergely Toldi et al.	2011	Hungary	103	PE vs. ctrl	IL6, CRP	PE	41	31 (26.5–34.5)^b^	35 (30.5–37.5)^b^	–	155 (140–185)^b^	95 (85–110)^b^
						ctrl	62	31 (28–35)^b^	36 (33.5–38)^b^	–	105 (100–120)^b^	70 (60–80)^b^
Cristina Catarino et al.	2012	Portugal	88	PE vs. ctrl	IL-6, TNF, CRP	PE	46	29.7 ± 5.3^a^	at birth	29.8 (26.8; 32.8)^b^	155.1 ± 14.6^a^	97.1 ± 6.3^a^
						ctrl	42	30.4 ± 5.7^a^	at birth	29.8 (26.8; 32.8)^b^	119.9 ± 11.5^a^	69.0 ± 7.2^a^
Deniz Cemgil Arikan et al.	2012	Turkey	138	MPE vs. ctrl SEP vs. ctrl MEP vs. SPE	IL-4, IL-8, IL-12, IFN-γ, CRP	MPE	42	29.40±7.23^a^	33.55±1.45^a^	23.63±2.52^a^	154.76±5.05^a^	99.29±5.36^a^
						SPE	40	28.18±5.93^a^	33.00±1.13^a^	23.51±3.02^a^	181.88±23.74^a^	106.75±10.95^a^
						ctrl	56	28.46±4.44^a^	33.62±1.27^a^	23.03±2.71^a^	111.07±12.09^a^	68.66±9.79^a^
A. Ozler et al.	2012	Turkey	66	MPE vs. ctrl SEP vs. ctrl MEP vs. SPE	IL6 and TNF α	MPE	22	29.2 ± 5.8^a^	32.7 ± 3.6^a^	28.3 ± 4^a^	–	106.6 ± 7.7^a^
						SPE	20	30.3 ± 6.3^a^	34.3 ± 2.8^a^	31.1 ± 5^a^	–	91.1 ± 9.2^a^
						ctrl	24	32.3 ± 6.9^a^	34.7 ± 4.1^a^	28.2 ± 2.8^a^	–	76.9 ± 16^a^
J.P. Xiao et al.	2012	China	178	PE vs. ctrl	IL-6	PE	104	30 (18-40)^c^	33 (22–39)^c^	–	157 (129–186)^c^	108 (77–140)^c^
						ctrl	74	26 (19-38)^c^	36 (28–42)^c^	–	120 (90–138)^c^	76.5 (60–90)^c^
Danilla Michelle Costa e Silva et al.	2013	Brazil	90	MPE vs. ctrl SEP vs. ctrl MEP vs. SPE	IL-2, IL-6, IL-10, TNF	MPE	14	26.02 ± 6.98^a^	>24	–	161.10 ± 20.10^a^	107.80 ± 16.07^a^
						SPE	26		>24	–		
						ctrl	50	24.28 ± 4.97^a^	>24	–	119.10 ± 11.32^a^	79.96 ± 9.30^a^
Melina B. Pinheiro et al.	2014	Brazil	156	SPE vs. ctrl	IL-6, IL-8, TNF, IFN-γ	SPE	59	26 (21–29)^b^	–	23.2 (21.4–28.4)^b^	160 (160-180)^b^	110 (100–115)^b^
						ctrl	49	23 (18–29)^b^	–	23.3 (20.9–26.9)^b^	110 (100–110)^b^	70 (63–70)^b^
Muzaffer Cakmak et al.	2015	Turkey	129	PE vs. ctrl	TNF	PE	99	28.6 ± 6.5^a^	34.5 ± 3.8^a^	28.31 ± 7.28^a^	152 ± 14^a^	98 ± 10^a^
						ctrl	30	27.2 ± 7.9^a^	34.2 ± 2.5^a^	27.07 ± 6.17^a^	103 ± 5^a^	62 ± 4^a^
Kelly K. Ferguson et al.	2017	US	441	PE vs. ctrl	IL-1β, IL-6, IL-10, TNF, RCP	PE	50	–	18/26/35	–	–	–
						ctrl	391	–	18/26/35	–	–	–
Jorge Valencia-Ortega et al.	2018	Mexico	100	PE vs. ctrl	TNF, IL-6, IL-8, IL-10, and IL-1RA	PE	50	28.5 (23.0–34.3)^b^	–	26.5 (23.9–30.0)^b^	142.0 (122.0–154.0)^b^	87.0 (72.0–97.0)^b^
						ctrl	50	28.0 (23.0–32.0)^b^	–	25.5 (22.5–29.3)^b^	116.0 (109.8–121.3)^b^	70.0 (66.5–76.3)^b^
Ayse Ekin Kara et al.	2019	Turkey	56	PE vs. ctrl	IL-6, CRP	PE	20	30 ± 5.3^a^	–	30.2 ± 5.6^a^	150 ± 11.1^a^	110 ± 10^a^
						ctrl	36	30 ± 5.3^a^	–	29.4 ± 1.8^a^	90 ± 13^a^	70 ± 7.4^a^
Yan-hua Liu et al.	2022	China	932	PE vs. ctrl	TNF, IFN-γ, TGF- α, IL-2, IL-4, IL-10, IL-17A	PE	466	30.88 ± 5.00^a^	34.3 ± 2.9^a^	29.4 ± 1.8^a^	–	–
						ctrl	466	31.00 ± 4.58^a^	34.4 ± 2.7^a^	29.4 ± 1.8^a^	–	–

[N, number; BMI, body mass index (kg/m^2^); SBP, systolic blood pressure (mmHg); DBP, diastolic blood pressure (mmHg); PE, pre-eclampsia; MPE, mild pre-eclampsia; SPE, severe pre-eclampsia; ctrl, control]. Data are presented as: [^a^mean ± standard deviation; ^b^median (25th–75th percentile); ^c^median (range)].

The participants were recruited from several countries. Most patients and controls were between 25 and 30 years old, with BMI ranging from 23 to 31 kg/m^2^. All samples were collected after 24 weeks of gestation. The mean blood pressure was 153/98 mm Hg in the PE group and 113/71 mm Hg in the control group.

Sample characteristics are displayed in **Supplementary Table 3**. We recorded the CRP, IL-6, IL-8, TNF, IL-4, and IL-10 levels from each study. The samples were either serum or plasma, and the assay methods included enzyme-linked immunosorbent assays, multiplex analyses, cytometric bead arrays, and immunoturbidimetric assays.

### CRP analysis

**Figure 2:**
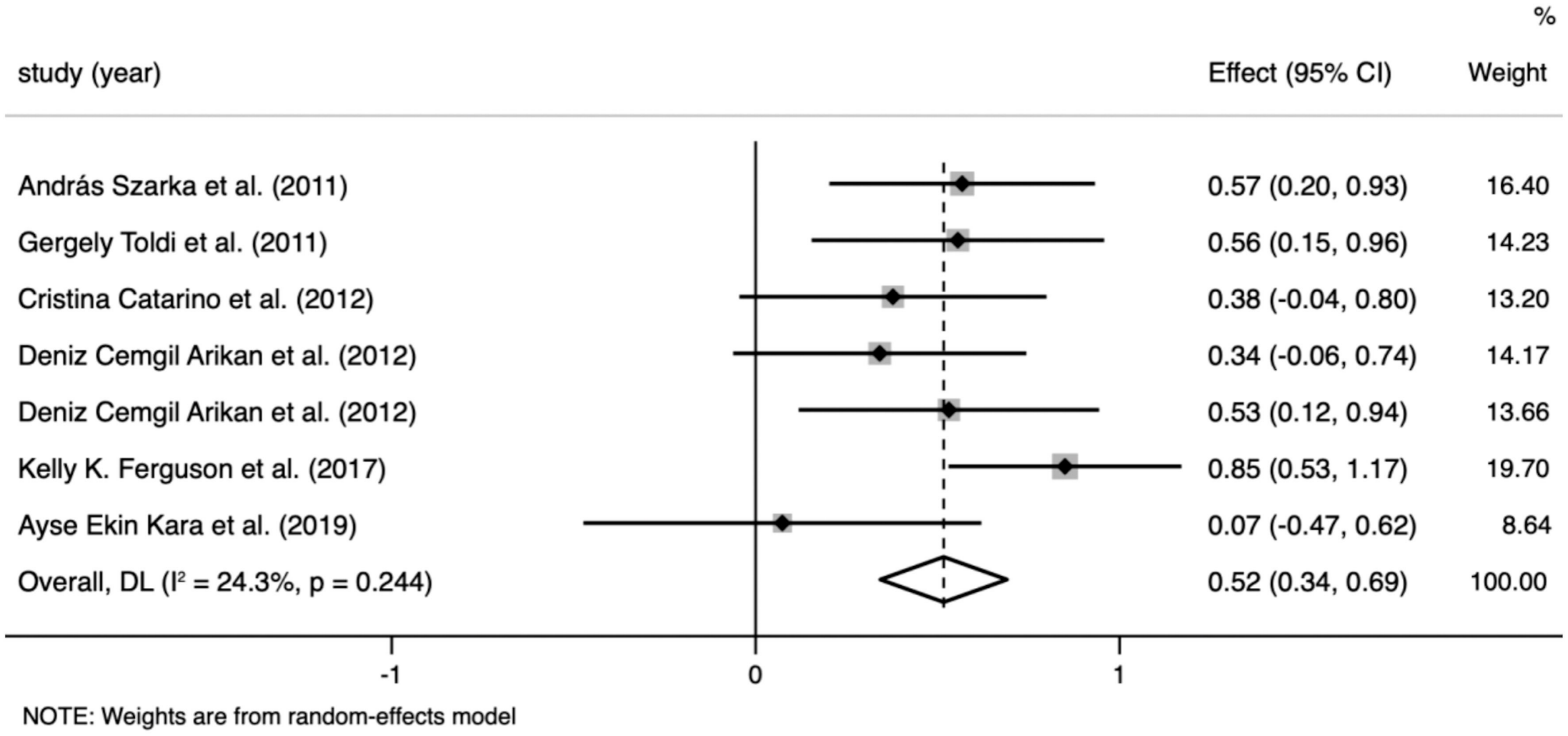
Meta-analysis of the levels of C-reactive protein (CRP) in the pre-eclampsia and control groups. Forest plot of the overall analysis of CRP. Data are presented as standard mean difference (SMD) and 95% confidence interval (CI). SMD>0 indicates that the CRP level was higher in the pre-eclampsia group.

CRP levels were extracted from six studies that included 885 participants. The random-effects meta-analysis revealed a significant difference in CRP levels between the PE and control groups. CRP levels were significantly higher in patients with PE than in the controls (SMD=0.517; 95% CI, 0.343–0.690; p<0.001; **Figure 2**). The SMD showed no significant heterogeneity within the group (I² = 24.3%; p=0.244).

### Pro-inflammatory cytokine analysis

**Figure 3:**
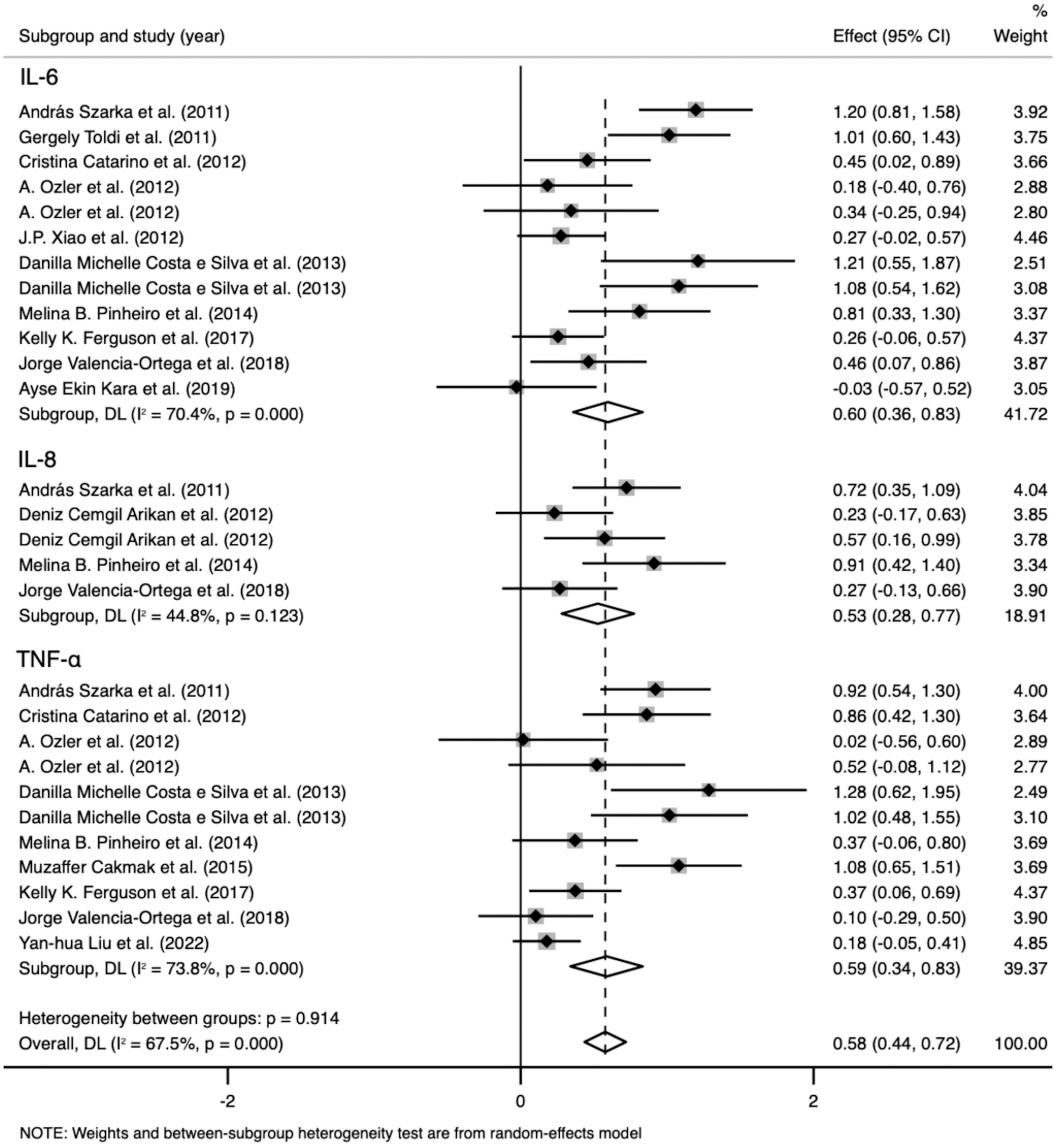
Meta-analysis of the pro-inflammatory cytokine levels in the pre-eclampsia and control groups. Forest plot of the overall analysis of interleukin (IL)-6, IL-8, and tumor necrosis factor (TNF). Data are presented as standard mean difference (SMD) and 95% confidence interval (CI). SMD>0 indicates that the biomarker level was higher in the pre-eclampsia group.

IL-6 levels were extracted from 10 studies that included 1,236 participants. The random-effects meta-analysis showed a significant difference between the PE and control groups. IL-6 levels were significantly higher in patients with PE than in the controls (SMD=0.596; 95% CI, 0.359–0.833; p<0.001; **Figure 3**). The SMD showed significant heterogeneity within the group (I²=70.4%; p<0.001). Subgroup analysis by GA reduced the heterogeneity (GA ≤ 34 weeks: I²=8.4%; p=0.359 vs. GA > 34 weeks: I²=0%; p=0.923 vs. at birth: I²=0%; p=0.997) (described in the subgroup analysis section for all cytokines).

TNF levels were extracted from nine studies that included 1,331 participants. The random-effects meta-analysis revealed a significant difference between the PE and control groups. TNF levels were significantly higher in patients with PE than in the controls (SMD=0.586; 95% CI, 0.339–0.833; p<0.001; **Figure 3**). The SMD showed significant heterogeneity within the group (I²=73.8%; p<0.001). Subgroup analysis by GA reduced the heterogeneity (GA ≤ 34 weeks: I²=0%; p=0.625 vs. GA > 34 weeks: I²=0%; p=0.818 vs. at birth: I²=84.3%; p=0.012).

IL-8 levels were extracted from four studies that included 431 participants. The random-effects meta-analysis demonstrated a significant difference between the PE and control groups. IL-8 levels were significantly higher in patients with PE than in the controls (SMD=0.527; 95% CI, 0.280–0.774; p<0.001; **Figure 3**). The SMD showed no significant heterogeneity within the group (I²=44.8%; p=0.123). Subgroup analysis by SBP reduced the heterogeneity (SBP < 160 mmHg: I²=0%; p=0.898 vs. SBP ≥ 160 mmHg: I²=0%; p=0.587).

### Anti-inflammatory cytokine analysis

**Figure 4:**
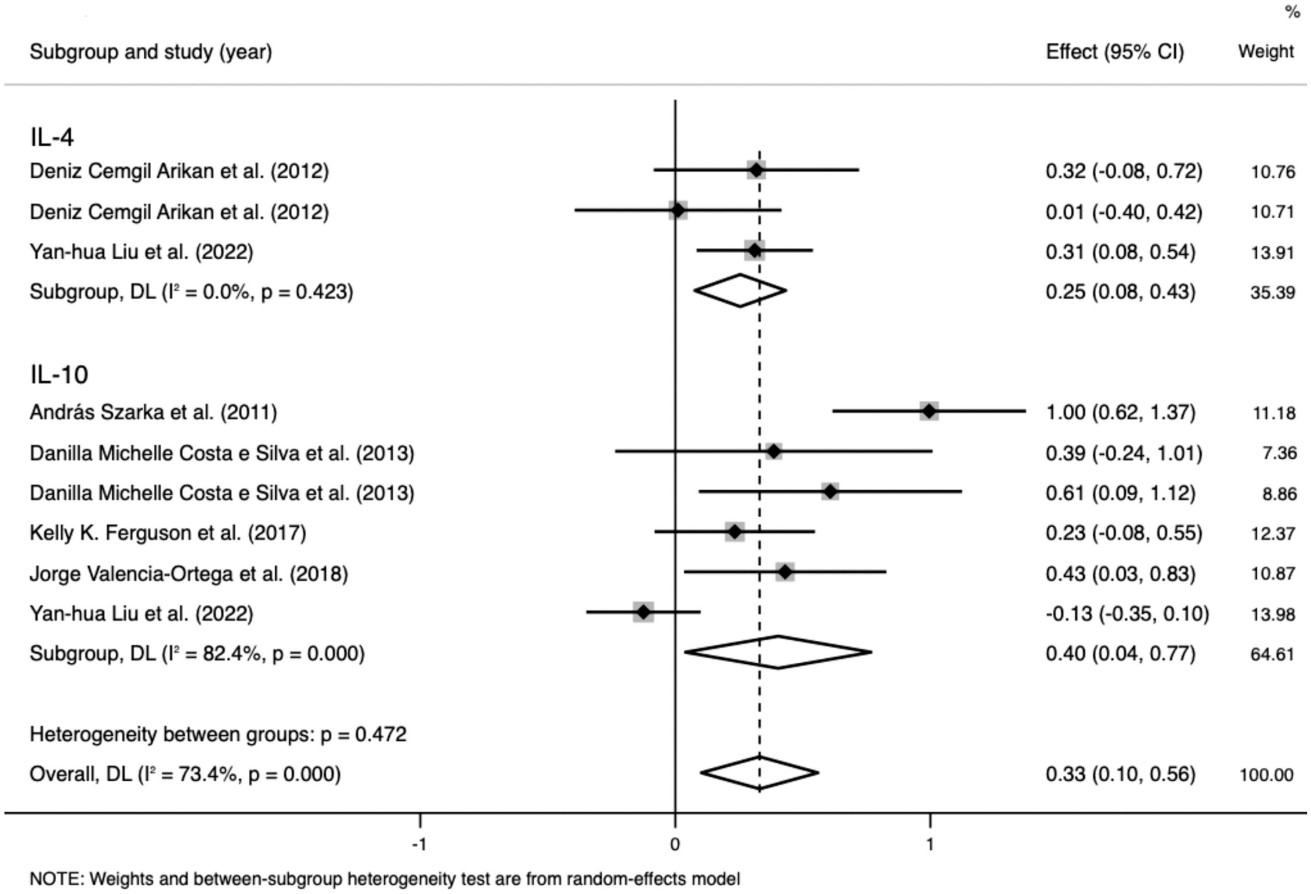
Meta-analysis of the levels of anti-inflammatory cytokines in the pre-eclampsia and control groups. Forest plot of the overall analysis of interleukin (IL)-6 and IL-10. Data are presented as standard mean difference (SMD) and 95% confidence interval (CI). SMD>0 indicates that the biomarker level was higher in the pre-eclampsia group.

Data on IL-4 levels were extracted from two studies that included 437 participants. The random-effects meta-analysis demonstrated a significant difference between the PE and control groups. IL-4 levels were significantly higher in patients with PE than in the controls (SMD=0.254; 95% CI, 0.076–0.433; p=0.005; **Figure 4**). The SMD revealed no significant heterogeneity within the group (I²=0%; p=0.423).

Data on IL-10 levels were extracted from five studies that included 982 participants. The random-effects meta-analysis demonstrated a significant difference between the PE and control groups. IL-10 levels were significantly higher in patients with PE than in the controls (SMD=0.403; 95% CI, 0.039–0.767; p=0.03; **Figure 4**). The SMD showed significant heterogeneity within the group (I²=82.4%; p<0.001). Subgroup analysis by SBP reduced the heterogeneity (SBP < 160 mmHg: I²=0%; p=0.906 vs. SBP ≥ 160 mmHg: I²=28.9%; p=0.236).

### Overall biomarker comparison

**Figure 5:**
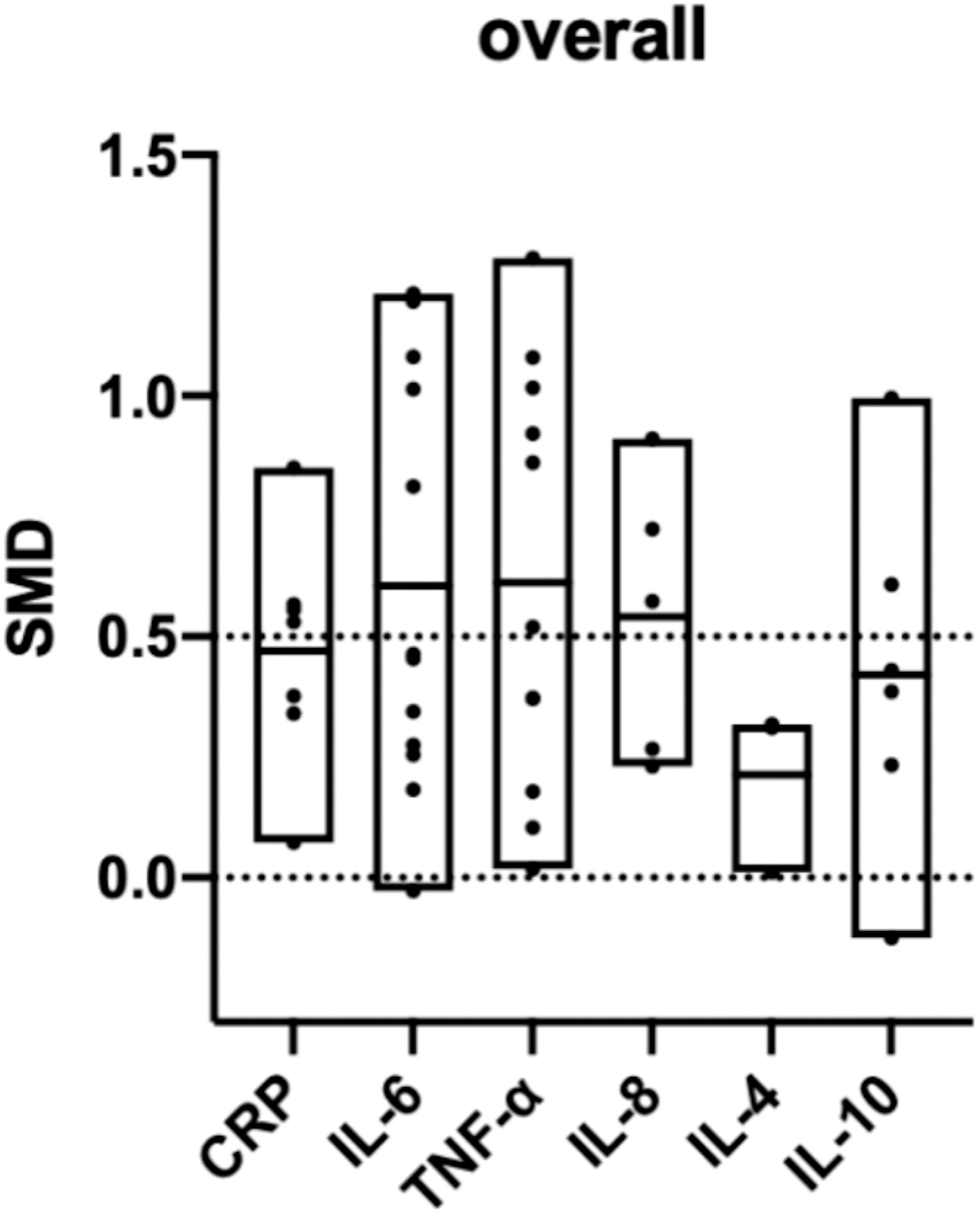
Overall comparison of the included biomarkers. (SMD: standard mean difference; CRP: C-reactive protein; IL: interleukin; TNF: tumor necrosis factor). SMD>0 indicates that the biomarker level was higher in the pre-eclampsia group. The middle line of each column represents the average SMD of each biomarker.

All six biomarker differences between the PE and control groups were significant. The inflammatory biomarkers were significantly higher in patients with PE than in the controls, and the SMDs were above 0 (**Table 2**). A cross-group comparison was performed between the biomarkers, and no significant differences were observed (p>0.05). However, we observed that the levels of pro-inflammatory biomarkers were significantly higher (SMD>0.5) than that of anti-inflammatory biomarkers (SMD<0.5) (**Figure 5**).

**Table 2:** SMD of included biomarkers.

	Random Effect SMD	95% CI	P Value
CRP	0.517	(0.343, 0.690)	p<0.001
pro-inflammatory cytokine			
IL-6	0.596	(0.359, 0.833)	p<0.001
TNF	0.586	(0.339, 0.833)	p<0.001
IL-8	0.527	(0.280, 0.774)	p<0.001
anti-inflammatory cytokine			
IL-4	0.254	(0.076, 0.433)	p=0.005
IL-10	0.403	(0.039, 0.767)	p=0.030

(SMD, standard mean difference; CI, confidence interval); p<0.05 was considered significant.

### Subgroup analysis by GA

Subgroup analysis by GA reduced the heterogeneity of IL-6 (GA ≤ 34 weeks: I²=8.4%; p=0.359 vs. GA > 34 weeks: I²=0%; p=0.923 vs. at birth: I²=0%; p=0.997) and TNF levels (GA ≤ 34 weeks: I²=0%; p=0.625 vs. GA > 34 weeks: I²=0%; p=0.818 vs. at birth: I²=84.3%; p=0.012), whereas the reduction was insignificant in the other groups (**Table 3**).

**Table 3:** Subgroup analysis by GA.

	Random Effect SMD (95% CI)	p for Effect Size	I² (%)	p for Heterogeneity	p for Cross Group Comparison
CRP					
Before	0.517 (0.343-0.690)	p<0.001	24.30%	p=0.244	
≤34	0.595 (0.288-0.903)	p<0.001	50.00%	p=0.135	p=0.358
>34	0.562 (0.292-0.832)	p<0.001	0.00%	p=0.966	
at birth	0.377 (-0.045-0.799)	p=0.080	–	–	
IL-6					
Before	0.596 (0.359-0.833)	p<0.001	70.40%	p<0.001	
≤34	0.343 (0.153-0.533)	p<0.001	8.40%	p=0.359	p<0.001
>34	1.118 (0.883-1.354)	p<0.001	0.00%	p=0.923	
at birth	0.459 (0.166-0.752)	p=0.002	0.00%	p=0.997	
TNF					
Before	0.586 (0.339-0.833)	p<0.001	73.80%	p<0.001	
≤34	0.265 (0.107-0.424)	p=0.001	0.00%	p=0.625	p<0.001
>34	1.031 (0.797-1.266)	p<0.001	0.00%	p=0.818	
at birth	0.476 (-0.266-01.217)	p=0.209	84.30%	p=0.012	
IL-8					
Before	0.527 (0.280-0.774)	p<0.001	44.80%	p=0.123	
≤34	0.551 (0.176-0.927)	p=0.004	55.60%	p=0.105	p=0.250
>34	0.723 (0.354-1.093)	p<0.001	–	–	
at birth	0.267 (-0.127-0.661)	p=0.184	–	–	
IL-10					
Before	0.403 (0.039-0.767)	p=0.030	82.40%	p<0.001	
≤34	0.036 (-0.313-0.385)	p=0.839	69.70%	p=0.069	p=0.026
>34	0.726 (0.366-1.086)	p<0.001	37.40%	p=0.202	
at birth	0.430 (0.034-0.827)	p=0.033	–	–	

[SMD, standard mean difference; CI, confidence interval; GA, gestational age (weeks)]. P<0.05 was considered significant; I²<50% indicated insignificant heterogeneity

**Figure 6:**
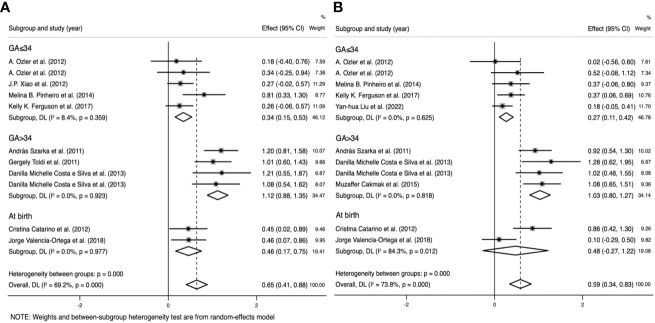
Subgroup analysis by gestational age **(GA)** (GA ≤ 34 weeks, GA>34 weeks, and at birth). **(A)** Forest plot of the subgroup analysis of interleukin-6. **(B)** Forest plot of the subgroup analysis of tumor necrosis factor. Data are presented as standard mean difference (SMD) and 95% confidence interval (CI). SMD>0 indicates that the biomarker level was higher in the pre-eclampsia group.

**Figure 7:**
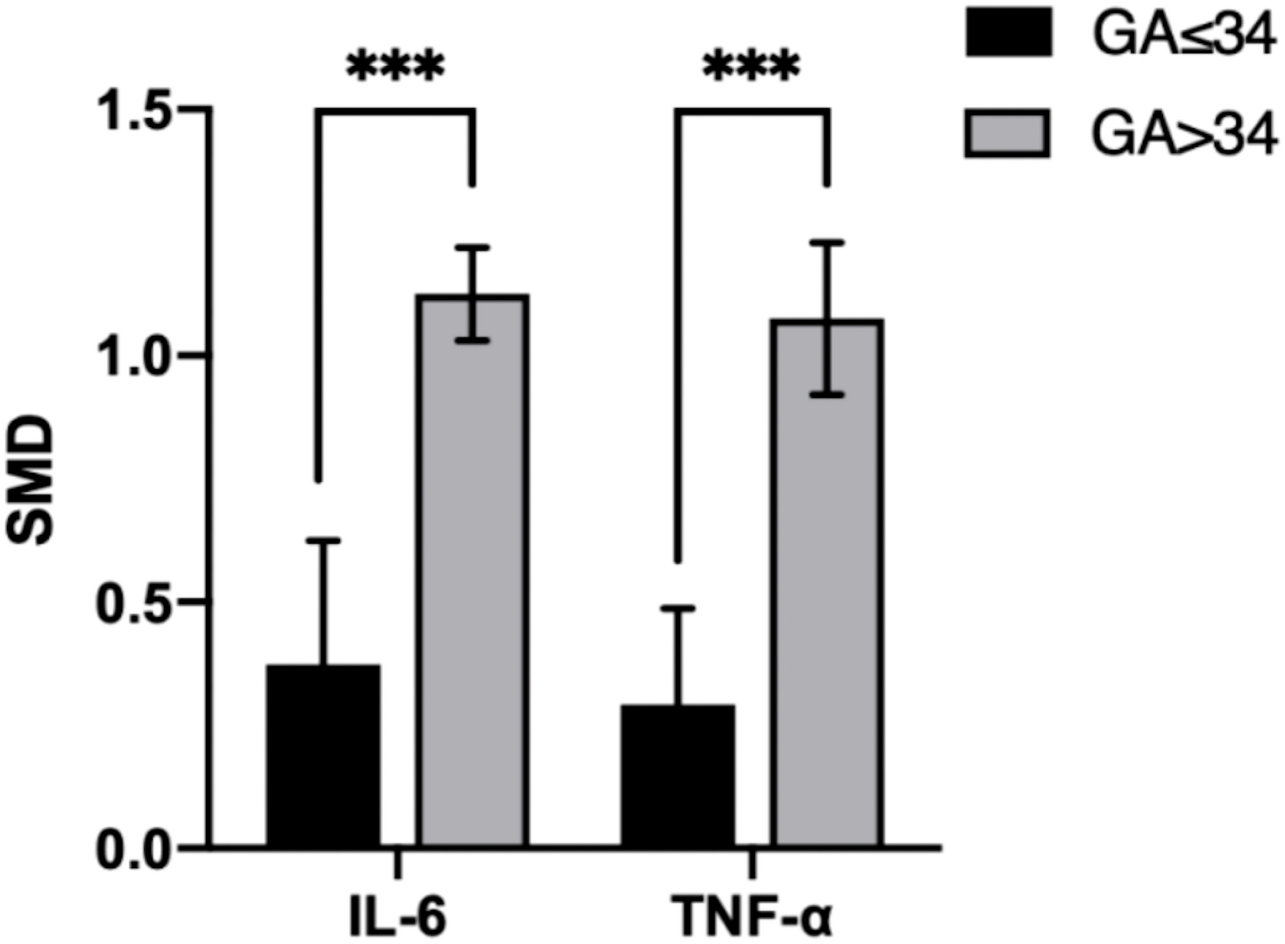
Cross-group comparison of GA. [GA: gestational age (weeks); SMD, standard mean difference; IL, interleukin; TNF, tumor necrosis factor]. Bar height represents the mean SMD of each biomarker. A higher bar indicates a more significant difference in biomarker levels between patients with pre-eclampsia and healthy pregnant women. ***p<0.001.

In the IL-6 group, five sample groups were obtained before 34 weeks of gestation, and four were obtained after 34 weeks. IL-6 levels were higher at GA > 34 weeks (SMD=1.118; 95% CI, 0.883–1.354; **Table 3**) than at GA ≤ 34 weeks (SMD=0.343; 95% CI, 0.153–0.533; **Table 3**). The cross-group comparison was significant (p<0.001; **Table 3**, **Figures 6A**, **7**).

In the TNF group, five sample groups were obtained before 34 weeks of gestation, and four were obtained after 34 weeks. IL-6 levels were higher at GA > 34 weeks (SMD=1.031; 95% CI, 0.797–1.266; **Table 3**) than at GA ≤ 34 weeks (SMD=0.265; 95% CI, 0.107–0.424; **Table 3**). The cross-group comparison was significant (p<0.001; **Table 3**, **Figures 6B**, **7**).

### Subgroup analysis by SBP

Subgroup analysis by SBP reduced the heterogeneity of IL-8 (SBP < 160 mmHg: I²=0%; p=0.898 vs. SBP ≥ 160 mmHg: I²=0%; p=0.587), IL-10 (SBP < 160 mmHg: I²=0%; p=0.906 vs. SBP ≥ 160 mmHg: I²=28.9%; p=0.236), and CRP levels (SBP < 160 mmHg: I²=0%; p=0.578 vs. SBP ≥ 160 mmHg: I²=0%; p=0.897), whereas the reduction was insignificant in the other groups (**Table 4**).

**Table 4:** Subgroup analysis by SBP.

	Random Effect SMD (95% CI)	p for Effect Size	I² (%)	p for Heterogeneity	p for Cross Group Comparison
CRP					
Before	0.517 (0.343-0.690)	p<0.001	24.30%	p=0.244	
<160	0.371 (0.154-0.588)	p=0.001	0.00%	p=0.578	p=0.051
≥160	0.551 (0.278-0.825)	p<0.001	0.00%	p=0.897	
IL-6					
Before	0.596 (0.359-0.833)	p<0.001	70.40%	p<0.001	
<160	0.539 (0.219-0.859)	p=0.001	68.80%	p=0.007	p<0.001
≥160	1.054 (0.789-1.318)	p<0.001	0.00%	p=0.476	
TNF					
Before	0.586 (0.339-0.833)	p<0.001	73.80%	p<0.001	
<160	0.803 (0.276-1.330)	p=0.003	80.40%	p=0.002	p=0.016
≥160	0.759 (0.366-1.152)	p<0.001	57.80%	p=0.094	
IL-8					
Before	0.527 (0.280-0.774)	p<0.001	44.80%	p=0.123	
<160	0.249 (-0.032-0.530)	p=0.082	0.00%	p=0.898	p=0.013
≥160	0.718 (0.477-0.958)	p<0.001	0.00%	p=0.587	
IL-10					
Before	0.403 (0.039-0.767)	p=0.030	82.40%	p<0.001	
<160	0.418 (0.083-0.752)	p=0.014	0.00%	p=0.906	p=0.008
≥160	0.842 (0.471-1.214)	p<0.001	28.90%	p=0.236	

[SMD, standard mean difference; CI, confidence interval; SBP, systolic blood pressure (mmHg)]. P<0.05 was considered significant; I²<50% indicated insignificant heterogeneity.

**Figure 8:**
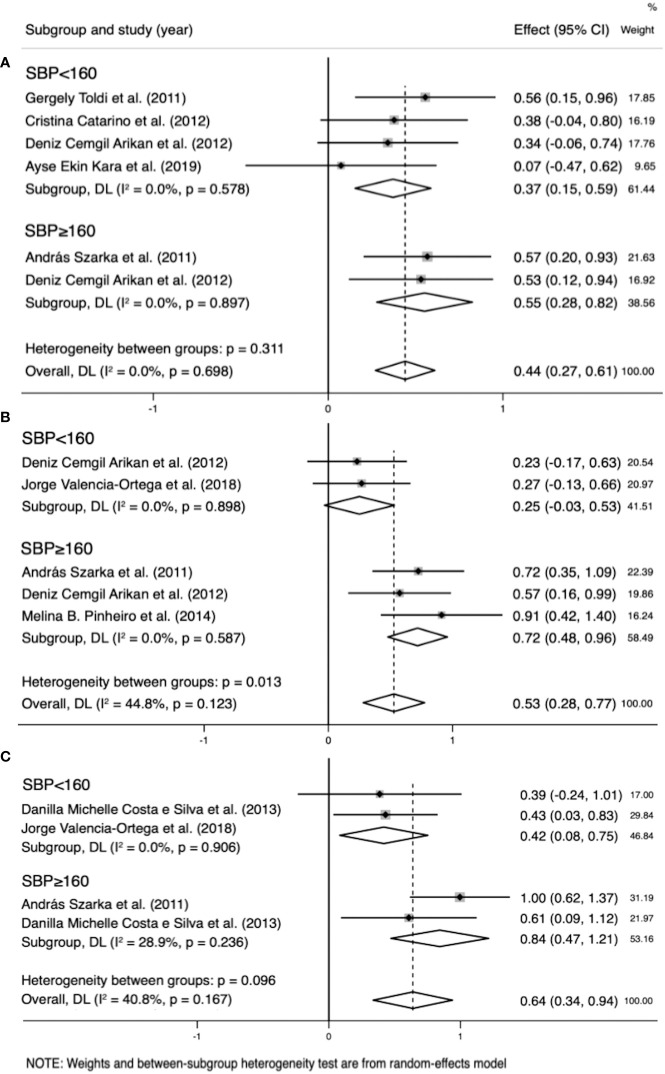
Subgroup analysis by systolic blood pressure (SBP) (SBP<160 mmHg, SBP≥160 mmHg). **(A)** Forest plot of the subgroup analysis of interleukin (IL)-8. **(B)** Forest plot of the subgroup analysis of IL-10. **(C)** Forest plot of the subgroup analysis of C-reactive protein. Data are presented as standard mean difference (SMD) and 95% confidence interval (CI). SMD>0 indicates that the biomarker level was higher in the pre-eclampsia group.

**Figure 9:**
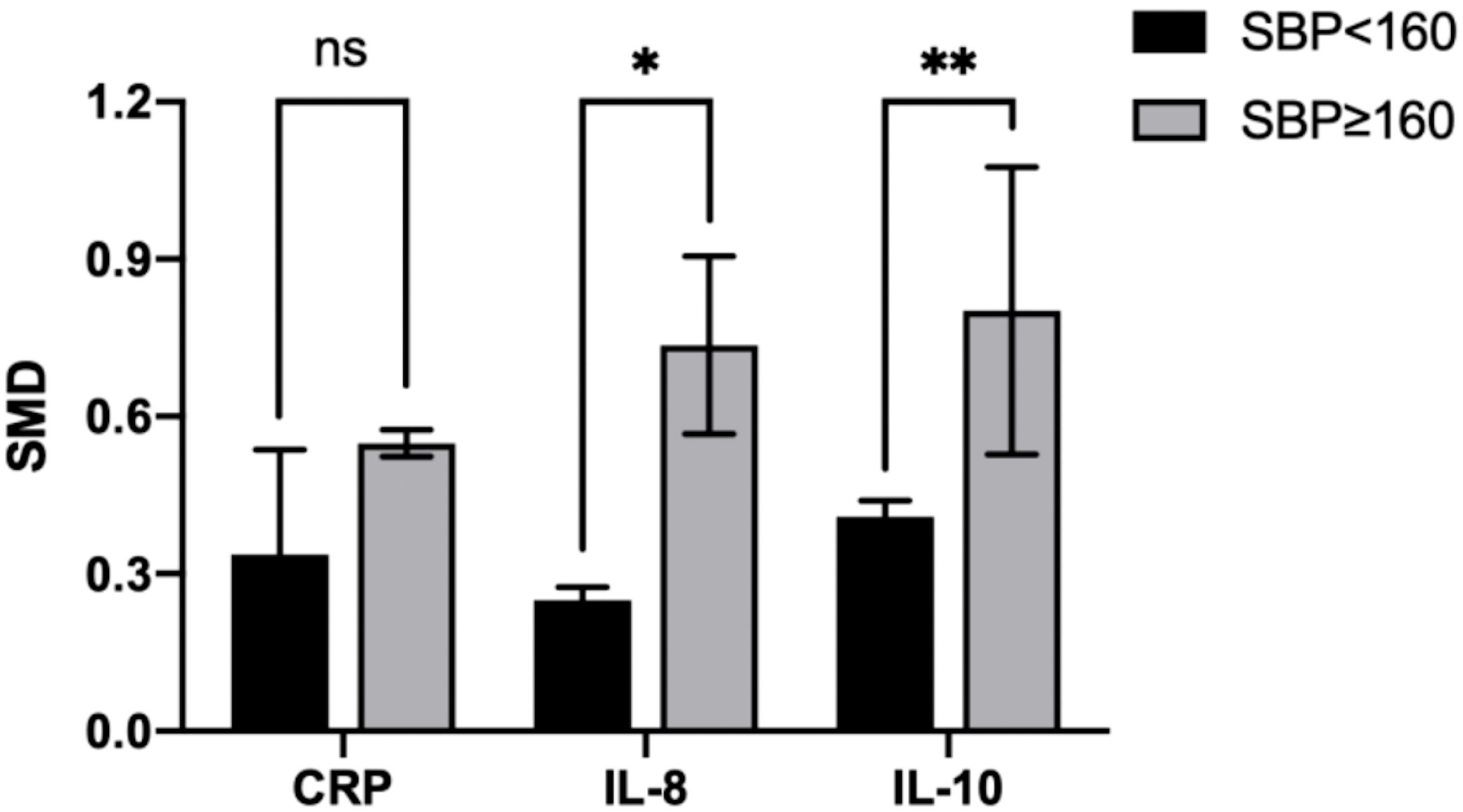
Cross-group comparison of SBP. [SBP: systolic blood pressure (mmHg); SMD, standard mean difference; CRP, C-reactive protein; IL, interleukin; ns, not significant]. Bar height represents the mean SMD of each biomarker. A higher bar indicates a more significant difference in biomarker levels between patients with pre-eclampsia and healthy pregnant women. *p<0.05; **p<0.01.

In the IL-8 group, the data of two groups of patients with SBP > 160 mmHg and three with SBP < 160 mmHg were analyzed. IL-8 levels were higher when SBP ≥ 160 mmHg (SMD=0.718; 95% CI, 0.477–0.958; **Table 4**) than SBP < 160 mmHg (SMD=0.249; 95% CI, -0.032–0.530; **Table 4**). The cross-group comparison was significant (p=0.013; **Table 4**; **Figures 8A**, **9**).

In the IL-10 group, the data of two groups of patients with SBP > 160 mmHg and two with SBP < 160 mmHg were analyzed. IL-10 levels were higher when SBP ≥ 160 mmHg (SMD=0.842; 95% CI, 0.471–1.214; **Table 4**) than SBP < 160 mmHg (SMD=0.418; 95% CI, 0.083–0.752; **Table 4**). The cross-group comparison was significant (p=0.008; **Table 4**; **Figures 8B**, **9**).

In the CRP group, the data of two groups of patients with SBP > 160 mmHg and four with SBP < 160 mmHg were analyzed. CRP levels were higher when SBP ≥ 160 mmHg (SMD=0.551, 95% CI, 0.278–0.825; **Table 4**) than SBP < 160 mmHg (SMD=0.249; 95% CI, 0.154–0.588; **Table 4**). The cross-group comparison was insignificant (p=0.051; **Table 4**; **Figures 8C**, **9**).

### Subgroup analysis by other relevant factors

We performed other subgroup analyses based on the test method (enzyme-linked immunosorbent assay, multiplex array, etc.), sample type (serum, plasma), age (< 30 years, ≥ 30 years), BMI (< 30 kg/m^2^, ≥ 30 kg/m^2^), and DBP (< 110 mmHg, ≥ 110 mmHg). Two of them slightly reduced the heterogeneity with a significant cross-group difference (**Supplementary Tables 4**, **5**), including age in the IL-10 group (age < 30 years: I²=40.8%; p=0.167 vs. age ≥ 30 years: I²=NA(not avaliable); p=NA; cross-group comparison p<0.001) and DBP in the CRP group (DBP < 110 mmHg: I²=0%; p=0.894 vs. DBP ≥ 110 mmHg: I²=NA; p=NA; cross-group comparison p=0.033). Other factors did not significantly reduce heterogeneity (**Supplementary Table 4**).

### Publication bias detection

Funnel plots for estimating publication bias were roughly symmetrical for all biomarkers (**Supplementary Figure 1**). No publication bias was detected by Begg’s test for IL-6 (p=0.409), TNF (p=0.099), IL-8 (p=0.495), IL-4 (p=0.586), or IL-10 (p=0.129). However, Begg’s test for CRP showed significant publication bias (p=0.003) (**Supplementary Figure 2**).

## Discussion

PE is a pregnancy complication associated with substantial maternal and fetal morbidity and mortality. Many theories about its underlying mechanism have been discussed, and inflammatory imbalance is now considered an important factor. Systemic conditions often influence the expression of inflammatory biomarkers, which may lead to inconsistencies in study results. A meta-analysis integrates information, eliminates the influence of interfering factors, and provides credible results. Further, because interfering factors influence biomarker levels, they directly or indirectly affect PE development and progression. In this study, we determined the relationship between inflammation and PE by comparing biomarker levels and identifying the factors involved in PE development and progression.

The balance between pro- and anti-inflammatory factors is crucial for placental implantation and pregnancy outcomes. Implantation elicits an inflammatory reaction, including the upregulation of IL-6 and TNF (41). In the early stages of normal pregnancy, a mild increase in the expression of T helper 1 (Th1) cytokines, the pro-inflammatory cytokines, is essential for the stimulation of new vessels for successful embryo implantation (42). However, prolonged exposure to Th1 cytokines may result in a cell-mediated immune response, which is harmful to the fetus (43). In particular, the expression of the Th2 cytokine IL-10 increases in the first trimester of pregnancy (44–46). Since IL-10 prevents the synthesis and secretion of various pro-inflammatory cytokines, it may be a protective mechanism of the human placenta in the first trimester of pregnancy to maintain trophoblast function and suppress inflammatory processes in the intervillous space (47). TNF levels during pregnancy increase in direct proportion to GA (48) and IL-10 levels are thought to increase until delivery (15). Studies have shown that natural killer cells and mast cells are key regulators of this inflammatory process; however, the function of cytokines remains unclear (49). Parturition is another key event associated with a pro-inflammatory environment, including the upregulation of IL-1b and IL-8 and the activation of the TNF signaling pathway (50). Smooth muscle contraction in the uterus can be induced by IL-1b, and cervical remodeling and dilation are accompanied by the infiltration of leukocytes into the cervix (49). As observed in the functions manifested during embryo implantation, uterine artery remodeling, and parturition, cytokines are essential to a successful pregnancy.

PE results from abnormal placentation due to insufficient trophoblast invasion and impaired spiral artery remodeling, followed by increased inflammatory and anti-angiogenic factors released from the placenta into the maternal circulation, leading to maternal syndrome (51). Cytokines that maintain the balance of Th1/Th2 cells during normal pregnancy are considerably involved in the pathogenesis of PE (52).

Our meta-analysis revealed increased levels of all included inflammatory biomarkers in patients with PE, especially CRP and pro-inflammatory cytokines. This result differs from that of a previous review that showed no differences in IL-8, IL-12, or IL-6 concentrations in maternal serum between women who later developed PE and healthy pregnant women (19). Thus, we deduced that other more important initiators contribute to PE development, and we focused on the anti-inflammatory cytokines.

Anti-inflammatory cytokines present at the maternal-fetal interface can regulate inflammatory responses, inhibit cellular immunity, regulate the invasion and differentiation of trophoblast cells, and induce placental growth and angiogenesis (53). In animal models, typical symptoms of PE, such as hypertension and proteinuria, can be present in mice without IL-4 (54). And lower concentrations of IL-10 can be detected in pregnant women who later develop PE (18, 19, 55). IL-4 can interact with progesterone, an IL-4 inducer, to downregulate the immune response of Th1 (54). IL-10 prevents the synthesis and secretion of various pro-inflammatory cytokines, such as TNF, IL-1β, IL-6, IL-8, and IL-12 (15, 56, 57). The lacking suppressive effects of these anti-inflammatory cytokines result in prolonged exposure to high levels of pro-inflammatory cytokines. Relatively high levels of pro-inflammatory cytokines stimulate the differentiation and activation of inflammatory cells in both the innate and adaptive arms of the immune system and may lead to immune activity against the fetus and vascular dysfunction (15). Excessive release of TNF may accelerate endothelial activation and injury, leading to PE symptoms (58). In pathological conditions of PE, necrotic trophoblasts increase IL-6 secretion, which triggers the activation of systemic endothelial cells. IL-6 is also heavily involved in the proliferation, invasion, and differentiation of trophoblast cells and oxidative stress in PE (59).

Our results revealed that the elevation of pro-inflammatory biomarkers is accompanied by an increase in the levels of anti-inflammatory cytokines, consistent with previous meta-analyses (60, 61). Macrophages, the major source of IL-10, are stimulated by TNF (62). We propose a new point of view that the elevation of the pro-inflammatory factors would activate the body’s defense system, leading to the production of anti-inflammatory cytokines. However, as shown by our results, the levels of anti-inflammatory cytokines though elevated, are still relatively lower than those of pro-inflammatory cytokines, resulting in PE progression.

PE can be divided into two subtypes based on GA (28–30). Our findings suggest significantly higher levels of IL-6 and TNF in patients sampled after 34 weeks of gestation. This finding can be explained in two ways. First, healthy pregnant women exhibited higher levels of these inflammatory biomarkers after 34 weeks of gestation. The immune system plays an important role in successful gestation. Implantation elicits an inflammatory reaction, including the upregulation of IL-6, and normal parturition may involve the activation of the TNF signaling pathway (41, 49, 50). Second, higher biomarker levels correlate with PE status. We suggest that the inflammatory balance in women at the late stage of pregnancy is more likely to be disrupted, and any precipitating factor would cause a rapid increase in biomarker levels, resulting in rapid onset and severe symptoms. However, other clinical studies showed more prominent biomarker changes in EOP than in LOP (22, 63). Further studies are warranted to resolve this discrepancy.

Perfect PE prediction remains elusive; however, a distinction between low- and high-risk PE is possible (64). Significant risk factors include previous PE or hypertension in pregnancy, chronic kidney disease, hypertension, type 1 or 2 diabetes, and autoimmune disorders (65, 66). Moderate risk factors are first pregnancy in women aged 40 years or more, a pregnancy interval greater than 10 years, BMI of 35 kg/m² or more, polycystic ovary syndrome, family history of PE, and multiple gestation (66, 67). These risk factors influence inflammatory biomarker levels. However, we observed no significant elevation of biomarker levels in patients with risk factors, including age ≥ 30 years and BMI ≥ 30 kg/m². Therefore, we hypothesized that a disturbance in the inflammatory balance is an independent risk factor for PE.

Remarkably, a higher gestational weight gain (GWG) is associated with a higher risk of gestational hypertensive disorders, including PE (68). Excessive weight gain also results in inflammatory imbalance. Previous studies have reported that excessive GWG results in higher IL-8 and CRP levels in pregnant women (69, 70). Given that pregnant women with excessive GWG have a higher probability of developing PE, the majority of the patients in the PE group are patients with GWG and higher inflammatory biomarkers, leading to selection bias. However, the causality between GWG and inflammatory status remains unclear; thus, further studies are required to clarify this relationship.

This study had some limitations. First, owing to the relatively small number of cohort studies, we narrowed our selection to case-control studies to reduce the inconsistencies in patient characteristics. The patients were diagnosed with PE according to the ACOG definition (2). Further, because the samples were obtained before diagnosis in the cohort studies, narrowing may result in a lack of data with predictive significance. Therefore, we compared the results of these studies with our results and discussed their similarities and differences. Second, the data on anti-inflammatory cytokines were insufficient, possibly resulting in an incomplete analysis.

In conclusion, an inflammatory imbalance is highly correlated with PE development. The impairment of the anti-inflammatory system is an initiating event that leads to the enhanced effect of the pro-inflammatory system. Higher levels of pro-inflammatory factors can activate the immune system; however, continuous disturbance of the inflammatory balance results in PE signs and symptoms. This disturbance of inflammatory balance is an independent risk factor for PE. Pregnant women after 34 weeks of gestation are more susceptible to PE. These results provide a basis for further research on the mechanisms of PE. We hope that more cohort studies will focus on the anti-inflammatory system.

## Author contributions

XG designed the program, searched and reviewed studies, were in charge of the manuscript. YF, YL, MC, assessed studies, extracted data. CZ, QZ, CL, JZ, CW and JS reviewed and edited the manuscript. JD had full access over all data in the study and is ultimately responsible for the decision to submit and publish the final version. All authors contributed to the article and approved the submitted version. All authors contributed to the article and approved the submitted version.

## Supplementary material

Supplementary Figure 1 Egger’s publication bias plot. (A) Egger’s test of C-reactive protein (CRP); (B) Egger’s test of interleukin (IL)-6; (C) Egger’s test of IL-8; (D) Egger’s test of tumor necrosis factor (TNF); (E) Egger’s test of IL-4; (F) Egger’s test of IL-10.

Supplementary Figure 2 Funnel plot of the publication bias. (A) Funnel plot of C-reactive protein (CRP); (B) Funnel plot of interleukin (IL)-6; (C) Funnel plot of IL-8; (D) Funnel plot of tumor necrosis factor (TNF); (E) Funnel plot of IL-4; (F) Funnel plot of IL-10.
