# Supplementary figures and images for: The role of inflammatory biomarkers in the development and progression of pre-eclampsia: a systematic review and meta-analysis
Source: Front Immunol. 2023 May 30;14:1156039. doi: 10.3389/fimmu.2023.1156039 (PMC10266420; doi:10.3389/fimmu.2023.1156039)

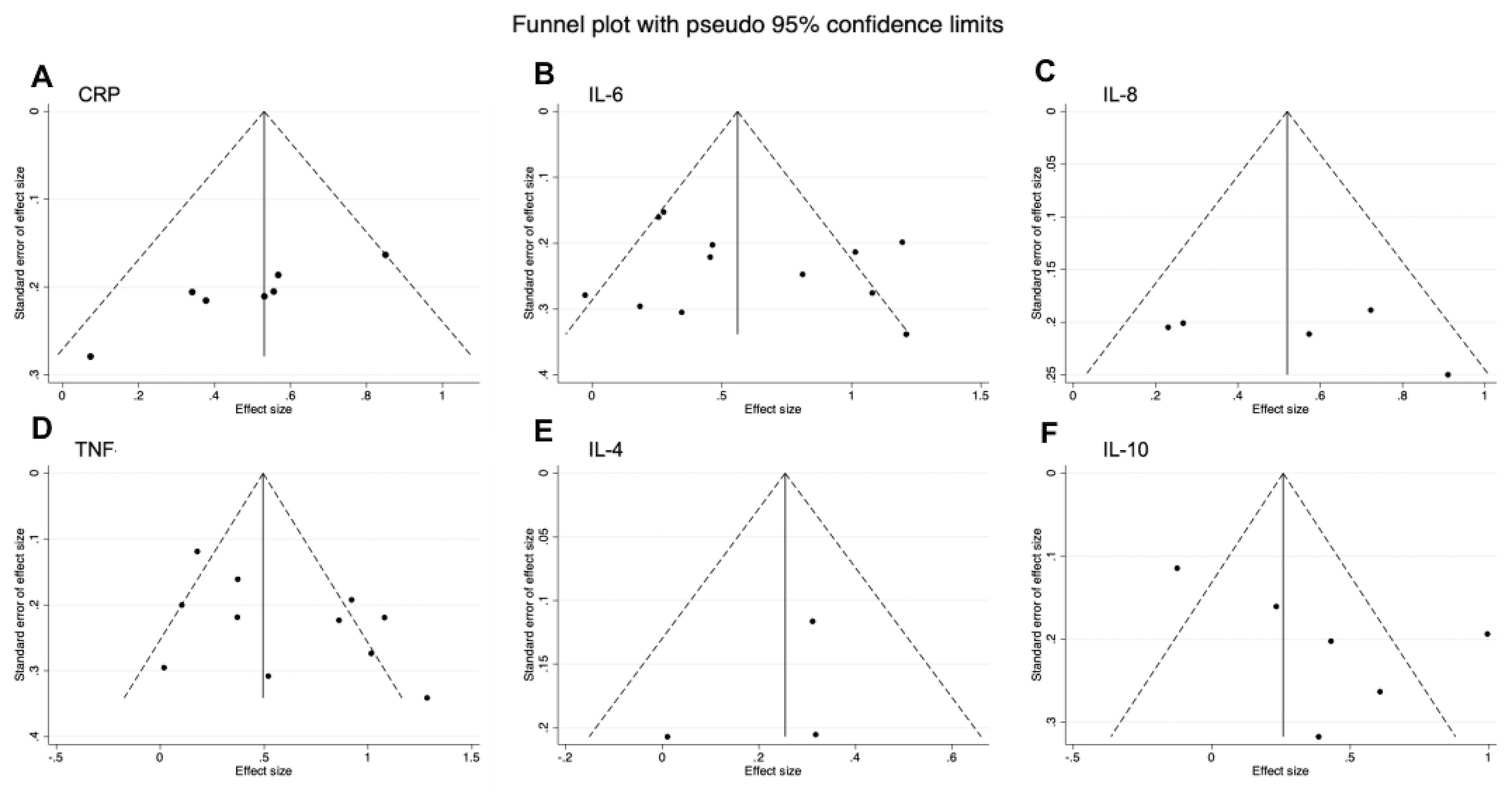

Supplement: Supplementary Figure 1 — Egger’s publication bias plot. (A) Egger’s test of C-reactive protein (CRP); (B) Egger’s test of interleukin (IL)-6; (C) Egger’s test of IL-8; (D) Egger’s test of tumor necrosis factor (TNF); (E) Egger’s test of IL-4; (F) Egger’s test of IL-10. [file Image_1.jpeg]

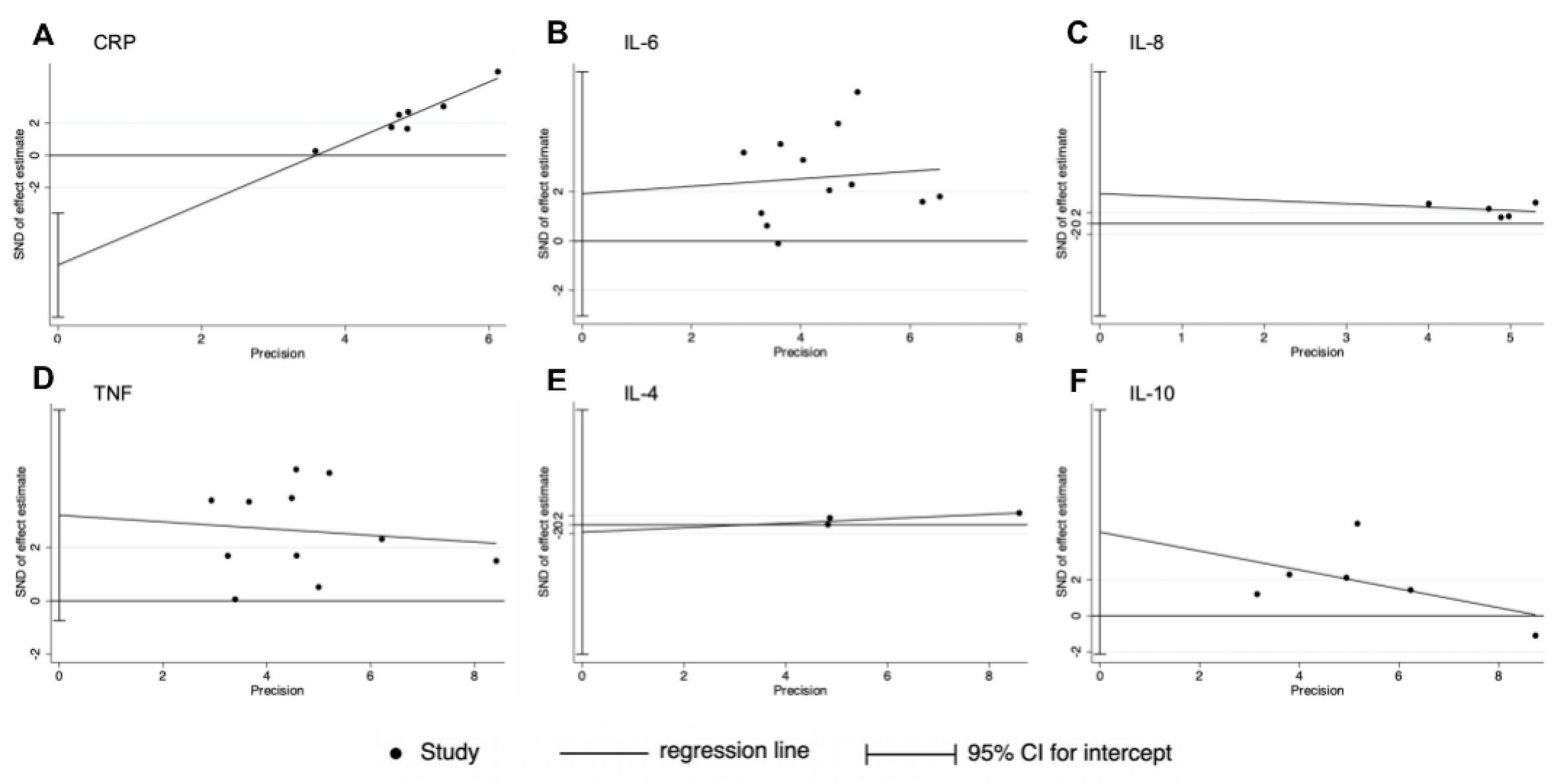

Supplement: Supplementary Figure 2 — Funnel plot of the publication bias. (A) Funnel plot of C-reactive protein (CRP); (B) Funnel plot of interleukin (IL)-6; (C) Funnel plot of IL-8; (D) Funnel plot of tumor necrosis factor (TNF); (E) Funnel plot of IL-4; (F) Funnel plot of IL-10. [file Image_2.jpeg]
