# Supplementary material for: The role of inflammatory biomarkers in the development and progression of pre-eclampsia: a systematic review and meta-analysis
Source: Front Immunol. 2023 May 30;14:1156039. doi: 10.3389/fimmu.2023.1156039 (PMC10266420; doi:10.3389/fimmu.2023.1156039)
Supplement: Supplementary file 3 [file Table_1.docx]

Supplementary Tables

# Supplementary Table 1 Search strategy

((("Pre-Eclampsia"[Mesh]) OR ((((((((((((((((((((((((((((((((((Pre Eclampsia) OR (Preeclampsia)) OR (Pregnancy Toxemias)) OR (Pregnancy Toxemia)) OR (Toxemia, Pregnancy)) OR (Edema-Proteinuria-Hypertension Gestosis)) OR (Edema Proteinuria Hypertension Gestosis)) OR (Gestosis, Edema-Proteinuria-Hypertension)) OR (Hypertension-Edema-Proteinuria Gestosis)) OR (Gestosis, Hypertension-Edema-Proteinuria)) OR (Hypertension Edema Proteinuria Gestosis)) OR (Toxemia Of Pregnancy)) OR (Of Pregnancies, Toxemia)) OR (Of Pregnancy, Toxemia)) OR (Pregnancies, Toxemia Of)) OR (Pregnancy, Toxemia Of)) OR (Toxemia Of Pregnancies)) OR (EPH Complex)) OR (EPH Toxemias)) OR (EPH Toxemia)) OR (Toxemia, EPH)) OR (Toxemias, EPH)) OR (EPH Gestosis)) OR (Gestosis, EPH)) OR (Toxemias, Pregnancy)) OR (Preeclampsia Eclampsia 1)) OR (1, Preeclampsia Eclampsia)) OR (1s, Preeclampsia Eclampsia)) OR (Eclampsia 1, Preeclampsia)) OR (Eclampsia 1s, Preeclampsia)) OR (Preeclampsia Eclampsia 1s)) OR (Proteinuria-Edema-Hypertension Gestosis)) OR (Gestosis, Proteinuria-Edema-Hypertension)) OR (Proteinuria Edema Hypertension Gestosis))) AND (("Inflammation"[Mesh]) OR ((((Inflammations) OR (Innate Inflammatory Response)) OR (Inflammatory Response, Innate)) OR (Innate Inflammatory Responses)))) AND ("cohort studies"[mesh] OR "case-control studies"[mesh] OR "comparative study"[pt] OR "risk factors"[mesh] OR "cohort"[tw] OR "compared"[tw] OR "groups"[tw] OR "case control"[tw] OR "multivariate"[tw])

# Supplementary Table 2 Quality assessment

| Study | Selection | Comparability | Exposure |
| --- | --- | --- | --- |
| András Szarka et al. | ★★ | ★★ | ★★★ |
| Gergely Toldi et al. | ★★ | ★★ | ★★★ |
| Cristina Catarino et al. | ★★ | ★★ | ★★★ |
| Deniz Cemgil Arikan et al. | ★★ | ★★ | ★★★ |
| A. Ozler et al. | ★★★ | ★★ | ★★★ |
| J.P. Xiao et al. | ★★★ | ★★ | ★★★ |
| Danilla Michelle Costa e Silva et al. | ★★ | ★★ | ★★★ |
| Melina B. Pinheiro et al. | ★★ | ★★ | ★★★ |
| Muzaffer Cakmak et al. | ★★★ | ★ | ★★★ |
| Kelly K. Ferguson et al. | ★★★ | ★★ | ★★★ |
| Jorge Valencia-Ortega et al. | ★★ | ★ | ★★★ |
| Ayse Ekin Kara et al. | ★★★ | ★ | ★★★ |
| Yan-hua Liu et al. | ★★★ | ★★ | ★★ |

# Supplementary Table 3 Sample characteristics

|  | Author | Year | Assay Methods | Sample type |  | N | Mean | SD |
| --- | --- | --- | --- | --- | --- | --- | --- | --- |
| CRP Article number: 6 Participant munber: 885 | | | | | | | | |
|  | András Szarka et al. | 2010 | Kits | Serum | PE | 60 | 7.22 | 7.14 |
|  |  |  |  |  | ctrl | 60 | 3.99 | 3.72 |
|  | Gergely Toldi et al. | 2011 | Immunoturbidimetric Assay | Plasma | PE | 41 | 8.29 | 9.87 |
|  |  |  |  |  | ctrl | 62 | 4.45 | 3.9 |
|  | Cristina Catarino et al. | 2012 | ELISA | Plasma | PE | 46 | 4.88 | 4.13 |
|  |  |  |  |  | ctrl | 42 | 3.45 | 3.38 |
|  | Deniz Cemgil Arikan et al. | 2012 | ELISA | Serum | MPE | 42 | 28.85 | 37.96 |
|  |  |  |  |  | SPE | 40 | 36.64 | 42.24 |
|  |  |  |  |  | ctrl | 56 | 16.67 | 33.95 |
|  | Kelly K. Ferguson et al. | 2017 | Multiplex Analysis | Plasma | PE | 44 | 9.59 | 9.95 |
|  |  |  |  |  | ctrl | 336 | 4.87 | 4.7 |
|  | Ayse Ekin Kara et al. | 2019 | Immunoturbidimetric Assay | Serum | PE | 20 | 5.1 | 6.2 |
|  |  |  |  |  | ctrl | 36 | 4.7 | 5 |
| Pro-inflammatory cytokines | | | | | | | | |
| IL-6 Article number: 10 Participant munber: 1236 | | | | | | | | |
|  | András Szarka et al. | 2011 | Multiplex Analysis | Serum | PE | 60 | 20.1 | 15.19 |
|  |  |  |  |  | ctrl | 60 | 7 | 3.04 |
|  | Gergely Toldi et al. | 2011 | Kits | Plasma | PE | 41 | 9.22 | 11.64 |
|  |  |  |  |  | ctrl | 62 | 1.72 | 1.29 |
|  | Cristina Catarino et al. | 2012 | ELISA | Plasma | PE | 42 | 5.56 | 5.98 |
|  |  |  |  |  | ctrl | 42 | 3.25 | 3.99 |
|  | A. Ozler et al. | 2012 | ELISA | Serum | MPE | 22 | 74.48 | 58.62 |
|  |  |  |  |  | SPE | 20 | 83.37 | 61.34 |
|  |  |  |  |  | ctrl | 24 | 64.78 | 47.05 |
|  | J.P. Xiao et al. | 2012 | ELISA | Serum | PE | 104 | 25.29 | 52.67 |
|  |  |  |  |  | ctrl | 74 | 13.95 | 13.62 |
|  | Danilla Michelle Costa e Silva et al. | 2013 | Multiplex Analysis | Plasma | MPE | 14 | 28.37 | 24.78 |
|  |  |  |  |  | SPE | 26 | 35.89 | 36.44 |
|  |  |  |  |  | ctrl | 36 | 8.79 | 11.43 |
|  | Melina B. Pinheiro et al. | 2014 | Cytometric Beads Array | Plasma | SPE | 43 | 17.42 | 14.17 |
|  |  |  |  |  | ctrl | 30 | 8.51 | 2.13 |
|  | Kelly K. Ferguson et al. | 2017 | Multiplex Analysis | Plasma | PE | 44 | 1.85 | 2 |
|  |  |  |  |  | ctrl | 336 | 1.51 | 1.22 |
|  | Jorge Valencia-Ortega et al. | 2018 | Multiplex Analysis | Serum | PE | 50 | 7.19 | 6.41 |
|  |  |  |  |  | ctrl | 50 | 4.82 | 3.36 |
|  | Ayse Ekin Kara et al. | 2019 | ELISA | Serum | PE | 20 | 3.7 | 20.1 |
|  |  |  |  |  | ctrl | 36 | 4.2 | 16.3 |
| IL-8 Article number: 4 Participant munber: 431 | | | | | | | | |
|  | András Szarka et al. | 2010 | Multiplex Analysis | Serum | PE | 60 | 131.69 | 180.78 |
|  |  |  |  |  | ctrl | 60 | 37.04 | 39.88 |
|  | Deniz Cemgil Arikan et al. | 2012 | ELISA | Serum | MPE | 42 | 6.54 | 18.46 |
|  |  |  |  |  | SPE | 40 | 13.55 | 25.73 |
|  |  |  |  |  | ctrl | 56 | 3.43 | 7.98 |
|  | Melina B. Pinheiro et al. | 2014 | Cytometric Beads Array | Plasma | SPE | 43 | 3.53 | 1.65 |
|  |  |  |  |  | ctrl | 30 | 2.35 | 0.41 |
|  | Jorge Valencia-Ortega et al. | 2018 | Multiplex Analysis | Serum | PE | 50 | 66.06 | 86.86 |
|  |  |  |  |  | ctrl | 50 | 45.46 | 65.95 |
| TNF Article number: 9 Participant munber: 1331 | | | | | | | | |
|  | András Szarka et al. | 2010 | Multiplex Analysis | Serum | PE | 60 | 2.35 | 0.76 |
|  |  |  |  |  | ctrl | 60 | 1.65 | 0.76 |
|  | Cristina Catarino et al. | 2012 | ELISA | Plasma | PE | 46 | 1.86 | 0.84 |
|  |  |  |  |  | ctrl | 42 | 1.27 | 0.46 |
|  | A. Ozler et al. | 2012 | ELISA | Serum | MPE | 22 | 8.93 | 4.52 |
|  |  |  |  |  | SPE | 20 | 12.41 | 8.22 |
|  |  |  |  |  | ctrl | 24 | 8.84 | 5.5 |
|  | Danilla Michelle Costa e Silva et al. | 2013 | Multiplex Analysis | Plasma | MPE | 14 | 11.57 | 5.74 |
|  |  |  |  |  | SPE | 26 | 10.59 | 5.03 |
|  |  |  |  |  | ctrl | 36 | 6.62 | 2.85 |
|  | Melina B. Pinheiro et al. | 2014 | Cytometric Beads Array | Plasma | SPE | 50 | 4.36 | 1.05 |
|  |  |  |  |  | ctrl | 37 | 4 | 0.85 |
|  | Muzaffer Cakmak et al. | 2015 | ELISA | Serum | PE | 99 | 26.49 | 12.14 |
|  |  |  |  |  | ctrl | 30 | 14.62 | 5.61 |
|  | Kelly K. Ferguson et al. | 2017 | Multiplex Analysis | Plasma | PE | 44 | 3.42 | 1.18 |
|  |  |  |  |  | ctrl | 336 | 2.92 | 1.36 |
|  | Jorge Valencia-Ortega et al. | 2018 | Multiplex Analysis | Serum | PE | 50 | 10.62 | 7.48 |
|  |  |  |  |  | ctrl | 50 | 9.88 | 6.79 |
|  | Yan-hua Liu et al. | 2022 | ELISA | Serum | PE | 139 | 9.4 | 10.95 |
|  |  |  |  |  | ctrl | 146 | 7.78 | 6.89 |
| Anti-inflammatory cytokines | | | | | | | | |
| IL-4 Article number: 2 Participant munber: 437 | | | | | | | | |
|  | Deniz Cemgil Arikan et al. | 2012 | ELISA | Serum | MPE | 42 | 0.9 | 0.85 |
|  |  |  |  |  | SPE | 40 | 0.59 | 0.65 |
|  |  |  |  |  | ctrl | 56 | 0.58 | 1.11 |
|  | Yan-hua Liu et al. | 2022 | ELISA | Serum | PE | 150 | 2.05 | 1.51 |
|  |  |  |  |  | ctrl | 149 | 1.64 | 1.09 |
| IL-10 Article number: 5 Participant munber: 982 | | | | | | | | |
|  | András Szarka et al. | 2010 | Multiplex Analysis | Serum | PE | 60 | 27.84 | 15.58 |
|  |  |  |  |  | ctrl | 60 | 16.54 | 3.89 |
|  | Danilla Michelle Costa e Silva et al. | 2013 | Multiplex Analysis | Plasma | MPE | 14 | 19.04 | 17.97 |
|  |  |  |  |  | SPE | 26 | 33.86 | 51.4 |
|  |  |  |  |  | ctrl | 36 | 11.94 | 18.55 |
|  | Kelly K. Ferguson et al. | 2017 | Multiplex Analysis | Plasma | PE | 44 | 15.41 | 9.29 |
|  |  |  |  |  | ctrl | 336 | 13.52 | 7.95 |
|  | Jorge Valencia-Ortega et al. | 2018 | Multiplex Analysis | Serum | PE | 50 | 16.59 | 18.47 |
|  |  |  |  |  | ctrl | 50 | 9.76 | 12.75 |
|  | Yan-hua Liu et al. | 2022 | ELISA | Serum | PE | 149 | 6.69 | 5.9 |
|  |  |  |  |  | ctrl | 157 | 7.74 | 10.22 |

# Supplementary Table 4 Results of subgroup analysis - heterogeneity reduction

|  |  | **I² (%)** | p for Heterogeneity |  | **I² (%)** | p for Heterogeneity |  | **I² (%)** | p for Heterogeneity |  | **I² (%)** | p for Heterogeneity |  | **I² (%)** | p for Heterogeneity |
| --- | --- | --- | --- | --- | --- | --- | --- | --- | --- | --- | --- | --- | --- | --- | --- |
|  |  | CRP | |  | IL-6 | |  | TNF | |  | IL-8 | |  | IL-10 | |
| Overall |  | 24.30% | p=0.244 |  | 70.40% | p<0.001 |  | 73.80% | p<0.001 |  | 44.80% | p=0.123 |  | 82.40% | p<0.001 |
|  |  |  |  |  |  |  |  |  |  |  |  |  |  |  |  |
| Subgroup analysis | | |  |  |  |  |  |  |  |  |  |  |  |  |  |
| Test Method |  |  |  |  |  |  |  |  |  |  |  |  |  |  |  |
| ELISA |  | 0.00% | p=0.793 |  | 0.00% | p=0.738 |  | 79.00% | p=0.001 |  | 26.20% | p=0.244 |  | -- | -- |
| Multiplex Array |  | -- | -- |  | 79.40% | p=0.001 |  | 76.80% | p=0.002 |  | 63.50% | p=0.098 |  | 58.70% | p=0.046 |
| Others |  | 24.30% | p=0.291 |  | 0.00% | p=0.534 |  | -- | -- |  | -- | -- |  | -- | -- |
|  |  |  |  |  |  |  |  |  |  |  |  |  |  |  |  |
| Gestation Age | |  |  |  |  |  |  |  |  |  |  |  |  |  |  |
| ≤34 |  | 50.00% | p=0.135 |  | 8.40% | p=0.359 |  | 0.00% | p=0.625 |  | 55.60% | p=0.105 |  | 69.70% | p=0.069 |
| >34 |  | 0.00% | p=0.966 |  | 0.00% | p=0.923 |  | 0.00% | p=0.818 |  | -- | -- |  | 37.40% | p=0.202 |
| at birth |  | -- | -- |  | 0.00% | p=0.997 |  | 84.30% | p=0.012 |  | -- | -- |  | -- | -- |
|  |  |  |  |  |  |  |  |  |  |  |  |  |  |  |  |
| Sample |  |  |  |  |  |  |  |  |  |  |  |  |  |  |  |
| Serum |  | 0.00% | p=0.459 |  | 74.10% | p=0.009 |  | 79.60% | p<0.001 |  | 32.00% | p=0.221 |  | 92.40% | p<0.001 |
| Plasma |  | 39.60% | p=0.191 |  | 67.30% | p=0.002 |  | 62.60% | p=0.030 |  | -- | -- |  | 0.00% | p=0.473 |
|  |  |  |  |  |  |  |  |  |  |  |  |  |  |  |  |
| Age |  |  |  |  |  |  |  |  |  |  |  |  |  |  |  |
| <30 |  | 0.00% | p=0.819 |  | 62.10% | p=0.015 |  | 72.60% | p=0.001 |  | -- | -- |  | 40.80% | p=0.167 |
| ≥30 |  | 48.50% | p=0.164 |  | 73.40% | p=0.010 |  | 6.80% | p=0.300 |  | -- | -- |  | -- | -- |
|  |  |  |  |  |  |  |  |  |  |  |  |  |  |  |  |
| BMI |  |  |  |  |  |  |  |  |  |  |  |  |  |  |  |
| <30 |  | 0.00% | p=0.819 |  | 66.80% | p=0.017 |  | 78.70% | p<0.001 |  | -- | -- |  | -- | -- |
| ≥30 |  | -- | -- |  | 0.00% | p=0.367 |  | -- | -- |  | -- | -- |  | -- | -- |
|  |  |  |  |  |  |  |  |  |  |  |  |  |  |  |  |
| SBP |  |  |  |  |  |  |  |  |  |  |  |  |  |  |  |
| <160 |  | 0.00% | p=0.578 |  | 68.80% | p=0.007 |  | 80.40% | p=0.002 |  | 0.00% | p=0.898 |  | 0.00% | p=0.906 |
| ≥160 |  | 0.00% | p=0.897 |  | 0.00% | p=0.476 |  | 57.80% | p=0.094 |  | 0.00% | p=0.587 |  | 28.90% | p=0.236 |
|  |  |  |  |  |  |  |  |  |  |  |  |  |  |  |  |
| DBP |  |  |  |  |  |  |  |  |  |  |  |  |  |  |  |
| <110 |  | 0.00% | p=0.894 |  | 70.20% | p=0.001 |  | 70.40% | p=0.001 |  | 32.00% | p=0.221 |  | -- | -- |
| ≥110 |  | -- | -- |  | 80.30% | p=0.024 |  | -- | -- |  | -- | -- |  | -- | -- |

# Supplementary Table 5 Results of subgroup analysis - effective size

|  | Random Effect SMD  (95% CI) | p for Effect Size | p for CGC | Random Effect SMD  (95% CI) | p for Effect Size | p for CGC | Random Effect SMD  (95% CI) | p for Effect Size | p for CGC | Random Effect SMD  (95% CI) | p for Effect Size | p for CGC | Random Effect SMD  (95% CI) | p for Effect Size | p for CGC |
| --- | --- | --- | --- | --- | --- | --- | --- | --- | --- | --- | --- | --- | --- | --- | --- |
|  | CRP | | | IL-6 | | | TNF | | | IL-8 | | | IL-10 | | |
| Overall | 0.517 ( 0.343-0.690 ) | p<0.001 |  | 0.596 ( 0.359-0.833 ) | p<0.001 |  | 0.586 ( 0.339-0.833 ) | p<0.001 |  | 0.527 ( 0.280-0.774 ) | p<0.001 |  | 0.403 ( 0.039-0.767 ) | p=0.030 |  |
|  |  |  |  |  |  |  |  |  |  |  |  |  |  |  |  |
| Subgroup analysis | |  |  |  |  | p=0.001 |  |  | p=0.563 |  |  | p=0.232 |  |  |  |
| Test Method | |  | p=0.082 |  |  |  |  |  |  |  |  |  |  |  | p=0.001 |
| ELISA | 0.416 ( 0.178-0.654 ) | p=0.001 |  | 0.270 ( 0.072-0.467 ) | p=0.008 |  | 0.534 ( 0.117-0.950 ) | p=0.012 |  | 0.398 ( 0.062-0.733 ) | p=0.020 |  | -0.125 ( -0.349-0.099 ) | p=0.275 |  |
| Multiplex Array | 0.850 ( 0.530-1.170 ) | p<0.001 |  | 0.807 ( 0.376-1.237 ) | p<0.001 |  | 0.686 ( 0.291-1.094 ) | p=0.001 |  | 0.500 ( 0.054-0.947 ) | p=0.028 |  | 0.531 ( 0.234-0.828 ) | p<0.001 |  |
| Others | 0.456 ( 0.183-0.728 ) | p=0.001 |  | 0.928 ( 0.611-1.245 ) | p<0.001 |  | 0.371 ( -0.058-0.800 ) | p=0.090 |  | 0.911 ( 0.421-1.400 ) | p<0.001 |  | -- | -- |  |
|  |  |  |  |  |  |  |  |  |  |  |  |  |  |  |  |
| Gestation Age | |  | p=0.358 |  |  | p<0.001 |  |  | p<0.001 |  |  | p=0.250 |  |  | p=0.026 |
| ≤34 | 0.595 ( 0.288-0.903 ) | p<0.001 |  | 0.343 ( 0.153-0.533 ) | p<0.001 |  | 0.265 ( 0.107-0.424 ) | p=0.001 |  | 0.551 ( 0.176-0.927 ) | p=0.004 |  | 0.036 ( -0.313-0.385 ) | p=0.839 |  |
| >34 | 0.562 ( 0.292-0.832 ) | p<0.001 |  | 1.118 ( 0.883-1.354 ) | p<0.001 |  | 1.031 ( 0.797-1.266 ) | p<0.001 |  | 0.723 ( 0.354-1.093 ) | p<0.001 |  | 0.726 ( 0.366-1.086 ) | p<0.001 |  |
| at birth | 0.377 ( -0.045-0.799 ) | p=0.080 |  | 0.459 ( 0.166-0.752 ) | p=0.002 |  | 0.476 ( -0.266-01.217 ) | p=0.209 |  | 0.267 ( -0.127-0.661 ) | p=0.184 |  | 0.430 ( 0.034-0.827 ) | p=0.033 |  |
|  |  |  |  |  |  |  |  |  |  |  |  |  |  |  |  |
| Sample | |  | p=0.270 |  |  | p=0.178 |  |  | p=0.328 |  |  | p=0.100 |  |  | p=0.834 |
| Serum | 0.425 ( 0.216-0.634 ) | p<0.001 |  | 0.428 ( 0.071-0.785 ) | p=0.019 |  | 0.473 ( 0.108-0.837 ) | p=0.011 |  | 0.454 ( 0.215-0.693 ) | p<0.001 |  | 0.421 ( -0.266-1.108 ) | p=0.230 |  |
| Plasma | 0.622 ( 0.341-0.903 ) | p<0.001 |  | 0.760 ( 0.434-0.087 ) | p<0.001 |  | 0.719 ( 0.385-1.053 ) | p<0.001 |  | 0.911 ( 0.421-1.400 ) | p<0.001 |  | 0.343 ( 0.096-0.589 ) | p=0.006 |  |
|  |  |  |  |  |  |  |  |  |  |  |  |  |  |  |  |
| Age |  |  | p=0.089 |  |  | p=0.065 |  |  | p=0.071 |  |  | -- |  |  | p<0.001 |
| <30 | 0.461 ( 0.262-0.660 ) | p<0.001 |  | 0.763 ( 0.466-1.060 ) | p<0.001 |  | 0.695 ( 0.381-1.010 ) | p<0.001 |  | -- | -- |  | 0.638 ( 0.337-0.939 ) | p<0.001 |  |
| ≥30 | 0.352 ( -0.115-0.819 ) | p=0.140 |  | 0.417 ( -0.018-0.851 ) | p=0.060 |  | 0.231 ( -0.011-0.473 ) | p=0.062 |  | -- | -- |  | -0.125 ( -0.349-0.099 ) | p=0.275 |  |
|  |  |  |  |  |  |  |  |  |  |  |  |  |  |  |  |
| BMI |  |  | p=0.083 |  |  | p=0.094 |  |  | p=0.609 |  |  | -- |  |  | -- |
| <30 | 0.461 ( 0.262-0.660 ) | p<0.001 |  | 0.643 ( 0.294-0.992 ) | p<0.001 |  | 0.507 ( 0.188-0.826 ) | p=0.002 |  | -- | -- |  | -- | -- |  |
| ≥30 | 0.073 ( -0.473-0.620 ) | p=0.793 |  | 0.141 ( -0.262-0.545 ) | p=0.492 |  | 0.520 ( -0.084-1.124 ) | p=0.091 |  | -- | -- |  | -- | -- |  |
|  |  |  |  |  |  |  |  |  |  |  |  |  |  |  |  |
| SBP |  |  | p=0.051 |  |  | p<0.001 |  |  | p=0.016 |  |  | p=0.013 |  |  | p=0.008 |
| <160 | 0.371 ( 0.154-0.588 ) | p=0.001 |  | 0.539 ( 0.219-0.859) | p=0.001 |  | 0.803 ( 0.276-1.330 ) | p=0.003 |  | 0.249 ( -0.032-0.530 ) | p=0.082 |  | 0.418 ( 0.083-0.752 ) | p=0.014 |  |
| ≥160 | 0.551 ( 0.278-0.825 ) | p<0.001 |  | 1.054 ( 0.789-1.318 ) | p<0.001 |  | 0.759 ( 0.366-1.152 ) | p<0.001 |  | 0.718 ( 0.477-0.958 ) | p<0.001 |  | 0.842 ( 0.471-1.214 ) | p<0.001 |  |
|  |  |  |  |  |  |  |  |  |  |  |  |  |  |  |  |
| DBP |  |  | p=0.033 |  |  | p=0.135 |  |  | p=0.041 |  |  | p=0.100 |  |  | -- |
| <110 | 0.480 ( 0.301-0.658 ) | p<0.001 |  | 0.682 ( 0.404-0.960 ) | p<0.001 |  | 0.720 ( 0.405-1.035 ) | p<0.001 |  | 0.454 ( 0.215-0.693 ) | p<0.001 |  | -- | -- |  |
| ≥110 | 0.073 ( -0.473-0.620 ) | p=0.793 |  | 0.401 ( 0.421-1.224 ) | p=0.339 |  | 0.371 ( -0.058-0.800 ) | p=0.090 |  | 0.911 ( 0.421-1.400 ) | p<0.001 |  | -- | -- |  |

# Supplementary Figures and Tables

For more information on Supplementary Material and for details on the different file types accepted, please see [here](https://www.frontiersin.org/guidelines/author-guidelines#supplementary-material).

## Supplementary Figures

**Supplementary Figure 1.** The figure legends are required to have the same font as the main text, 12 point normal Times New Roman, single spaced. Please use a single paragraph for each legend and prepare the figures keeping in mind the PDF layout.
